# A ligand-independent integrin β1 mechanosensory complex guides spindle orientation

**DOI:** 10.1038/ncomms10899

**Published:** 2016-03-08

**Authors:** Nicoletta I. Petridou, Paris A. Skourides

**Affiliations:** 1 Department of Biological Sciences, University of Cyprus, University Ave 1, New Campus, Nicosia 2109, Cyprus

## Abstract

Control of spindle orientation is a fundamental process for embryonic development, morphogenesis and tissue homeostasis, while defects are associated with tumorigenesis and other diseases. Force sensing is one of the mechanisms through which division orientation is determined. Here we show that integrin β1 plays a critical role in this process, becoming activated at the lateral regions of the cell cortex in a ligand-independent manner. This activation is force dependent and polar, correlating with the spindle capture sites. Inhibition of integrin β1 activation on the cortex and disruption of its asymmetric distribution leads to spindle misorientation, even when cell adhesion is β1 independent. Examining downstream targets reveals that a cortical mechanosensory complex forms on active β1, and regulates spindle orientation irrespective of cell context. We propose that ligand-independent integrin β1 activation is a conserved mechanism that allows cell responses to external stimuli.

## Abstract (web summary)

During cell division, the orientation of the mitotic spindle is sensitive to forces exerted on the cell cortex. Here Petridou and Skourides show that such cues are sensed by a mechanosensory complex established on force activated integrin β1 independent of ligand binding.

## Graphical abstract

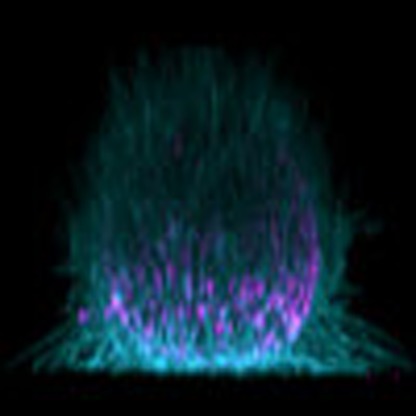

Spindle orientation is a fundamental process in all multicellular organisms important in both symmetrically and asymmetrically dividing cells. During asymmetric divisions, the spindle aligns parallel to a polarity axis so that cell fate determinants are asymmetrically inherited determining cell fate. In symmetric divisions like those of epithelial cells, the spindle is typically oriented parallel to the plane of the tissue, guiding tissue elongation, organ development and maintaining epithelial integrity^1^,^2^. The positioning and orientation of the mitotic spindle are achieved through the capture of astral microtubules (MTs) at discrete regions on the cell cortex via a conserved cortical complex (Gai/LGN/NuMA). The dynein/dynactin motor proteins are recruited at the cortex through interactions with this complex and exert pulling forces on astral MTs to position the spindle between the two capture sites^3^.

One of the more fascinating recent findings is that the spindle can respond to external mechanical forces. Specifically, evidence emerged that adherent cells sense forces transmitted through retraction fibres (RFs) and can dynamically reorient their spindles along force vectors^4^. Work in Zebrafish and *Xenopus* revealed that the same holds true in embryonic epithelia, where forces are presumably stemming from adherens and tight junctions that transmit tissue level tension^5^,^6^. However, our understanding of this process is lacking especially with respect to the proteins responsible for sensing such external stimuli.

Recent work from our group begun to unravel the molecular machinery responsible for force sensing in mitotic cells, when we showed that focal adhesion kinase (FAK)-null cells fail to orient their spindle in response to mechanical cues despite forming normal RFs^5^. FAK is a tyrosine kinase previously shown to be involved in mechanotransduction from integrin-based complexes called focal adhesions (FAs)^7^,^8^,^9^. Integrins, the transmembrane receptors that interact with extracellular matrix (ECM) components, undergo conformational changes on ligand binding that in turn induces the recruitment of interacting proteins and the formation of FAs linking the ECM to the actin cytoskeleton^10^. Integrin β1 has been identified as an important regulator of spindle orientation in cultured cells and in tissues, through its role in the maintenance of cell adhesion and the establishment of polarity in epithelia^11^,^12^,^13^,^14^,^15^,^16^,^17^,^18^. Surprisingly, however, depletion of FAK leads to defects in force sensing and spindle misorientation^5^,^19^ even in the *Xenopus* embryonic skin, where cells are not in contact with ECM^20^.

In this study, we show that integrin β1 becomes asymmetrically activated at the lateral cortex of mitotic cells and that both the activation and the asymmetric distribution of active β1 are critical for correct spindle orientation. We go on to show that this activation is ligand independent and force dependent. Examination of downstream effectors of integrin signalling revealed that the active forms of the FA proteins FAK, Src and p130Cas become enriched at the lateral cortex of mitotic cells in an integrin β1-dependent manner displaying similar asymmetric distributions. Finally, using rescue experiments in FAK- and Cas-null cells, we identify Cas as a regulator of spindle orientation and show that direct interactions of Cas and Src with FAK are critical for spindle orientation not only in adherent cells, but also in vertebrate epithelia.

## Results

### Integrin β1 is activated at the lateral mitotic cortex

**Figure 1:**
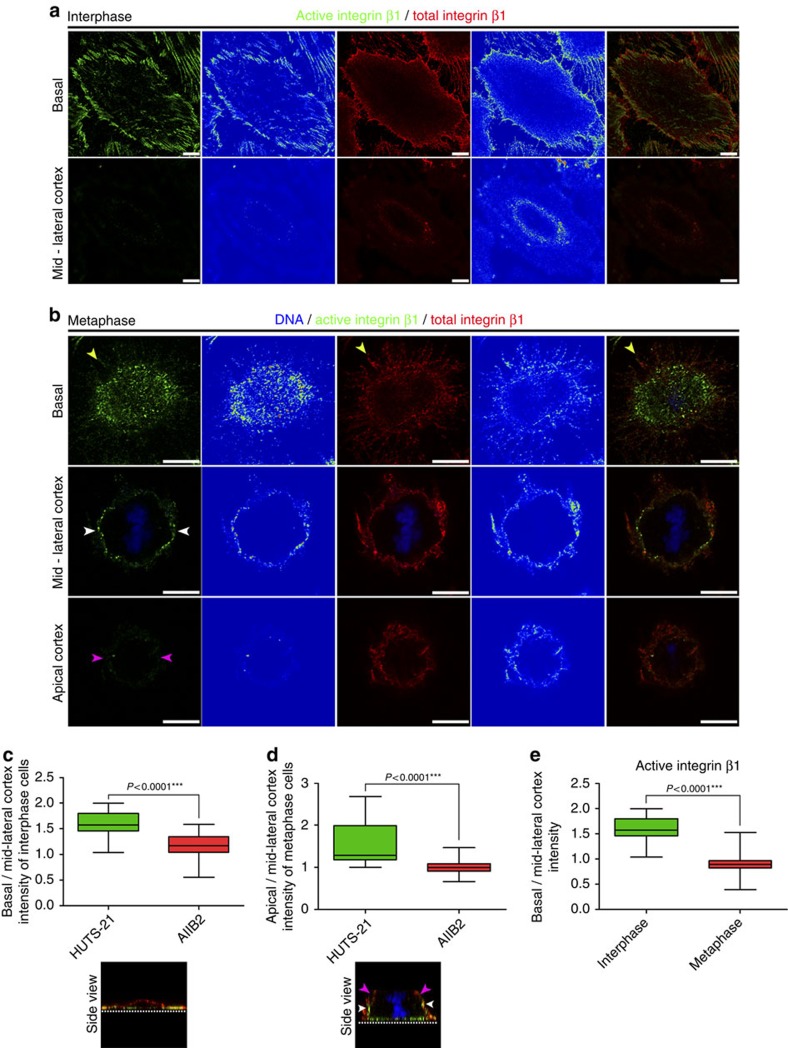
Integrin β1 becomes activated at the lateral cortex of mitotic cells. (**a**) Representative optical sections, intensity-coded and merged images at the cell–ECM interface and the lateral cortex of interphase HeLa cells stained for active (HUTS-21) and total (AIIB2) integrin β1. (**b**) Representative optical sections, colour intensity-coded and merged images at the cell–ECM interface, mid-lateral and apical areas of the cortex of metaphase cells stained as indicated. Yellow arrowheads show integrin β1-positive RFs. White arrowheads indicate active β1 at the lateral cortex at the plane of the spindle. Magenta arrowheads show absence of active β1 from the apical cortex. (**c**) Box-plot of the basal to mid-lateral cortex intensity ratio of active and total integrin β1 in interphase cells and a side view of the cell shown in **a** (dashed line shows the ECM). Mean±s.e.m.: HUTS-21 1.572±0.05921, *n*=20; AIIB2 1.158±0.05203, *n*=20; *P* values calculated by *t*-test; *n*, number of interphase cells, two independent experiments (one-way analysis of variance (ANOVA) for variability between replicates: HUTS-21 *P*=0.0643, ns, AIIB2 *P*=0.1955, ns). (**d**) Box-plot of the mid-lateral to apical cortex intensity ratio of active and total integrin β1 in metaphase cells and a side view of the cell shown in **b** (dashed line represents the ECM, white arrowheads indicate integrin β1 activation at the lateral cortex, magenta arrowheads show absence of active β1 at the apical areas of the cortex). Mean±s.e.m.: HUTS-21 1.523±0.1099, *n*=20; AIIB2 1.010±0.03892, *n*=20; *P* values calculated by *t*-test; *n*, number of metaphase cells, two independent experiments (one-way ANOVA: HUTS-21 *P*=0.5479, ns, AIIB2 p-0.2055, ns). (**e**) Box-plot of the basal to mid-lateral cortex intensity ratio of active integrin β1 in interphase and metaphase cells. Mean±s.e.m.: interphase 1.572±0.05921, *n*=20; metaphase 0.8887±0.04829, *n*=20; *P* values calculated by Mann–Whitney test; *n*, number of cells, two independent experiments (Kruskal–Wallis: interphase *P*=0.0643, ns, metaphase *P*=0.2879, ns). Scale bar, 10 μm (**a**,**b**) .

When cells in culture enter mitosis they round up and most of the FAs disassemble; however, cells retain RFs connecting them to the ECM through small adhesive complexes maintained at their terminations^5^,^21^. RFs have been shown to exert forces on the cell cortex and the mitotic spindle becomes aligned with such forces^4^. We have previously shown that in FAK-null cells RFs form normally, yet the spindle fails to respond to external forces^5^. This suggested that the adhesive complexes at RF terminations may signal to the cell, acting as mechanosensors. Since force application leads to integrin activation^22^,^23^, we decided to examine the distribution of active integrin β1 and asked whether it is enriched at RF terminations. To do so, we compared the distributions of active and total integrin β1 in HeLa cells, using two well-characterized antibodies, HUTS-21 (refs 24, 25) and AIIB2 (ref. 26), respectively. As shown, in interphase cells β1 is distributed uniformly throughout the cortex and in FA-like structures, while active β1 is found almost exclusively at FAs and is excluded from the lateral cortex (Fig. 1a,c). In mitotic cells, although β1 is distributed uniformly throughout the cell cortex and RFs, no enrichment of active β1 could be detected on RFs suggesting that the adhesive complexes act as mechanical anchoring points (Fig. 1b, yellow arrowheads). Surprisingly, we noted that active β1 is enriched at the lateral regions of the cell cortex at the plane of the spindle, a region not in contact with the substrate (Fig. 1b,d, white arrowheads), while it is absent from apical areas displaying strong apicobasal polarity (Fig. 1b,d,e magenta arrowheads). Activation of β1 at the cortex of mitotic cells was verified using an alternative combination of antibodies (Supplementary Fig. 1a,b) (refs 27, 28, 29).

**Figure 2:**
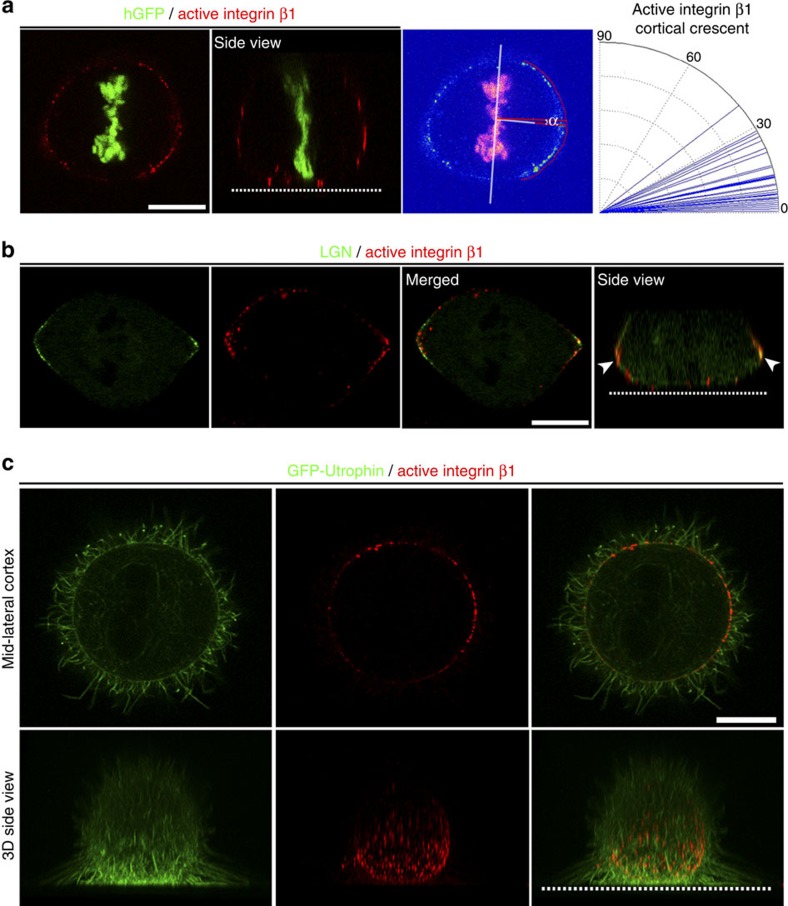
Active integrin β1 is distributed asymmetrically at the lateral cortex of mitotic cells. (**a**) A representative optical section and a side view of a live metaphase cell expressing histone GFP and stained for active integrin β1 (9EG7). Colour intensity image showing how the polarity crescent of active β1 was correlated with the spindle capture sites and an angular distribution plot of the angle α; mean±s.e.m.: 11.34±1.55°, *n*=35; n, number of metaphase cells, two independent experiments (two replicates each). (**b**) Labelling of active β1 in live metaphase cells expressing GFP–LGN and a side view showing active β1 at the cell–ECM interface and co-localization with LGN at the lateral cortex of the cell (white arrowheads). (**c**) Optical section at the mid-lateral cortex and a side view of a 3D reconstruction of a representative live metaphase cell expressing GFP–Utrophin stained for active β1 (9EG7) showing integrin activation at the areas where the RFs merge with the cell cortex. Scale bar, 10 μm (**a**–**c**).

In addition to the apicobasal polarity, integrin β1 activation was also polar at the plane of the spindle (Fig. 1b, white arrowheads). Live immunostaining of active integrin β1 gave a clearer signal with lower background making the polarity more pronounced. As shown, active β1 at the lateral regions of the cortex is distributed asymmetrically in metaphase cells with a polarity crescent accumulating at regions distal to the spindle poles (Fig. 2a). Furthermore, live immunostaining of active β1 in cells expressing green fluorescent protein (GFP)-leucine-glycine-asparagine repeat protein (LGN) showed that the localizations of LGN and active β1 are similar. Both display asymmetric distributions, preferentially accumulating at regions distal to the spindle (Fig. 2b, white arrowheads). Enrichment of active β1 at the lateral region of the cortex suggested that the distribution of active β1 may be determined by the distribution of RFs. High-resolution optical sections of live metaphase cells revealed a close association of RF terminations with active β1 and confirmed a correlation with the distribution of RFs. As shown, β1 displays the highest activation at areas where the RFs terminate on the cortex (Fig. 2c), a finding confirmed by staining fixed cells (Supplementary Fig. 2).

Overall, these data suggest that integrin β1 becomes asymmetrically activated at the lateral cortex during mitosis correlating with the distribution of RFs around the cell cortex.

### Integrin β1 activation on the cortex depends on RFs

**Figure 3:**
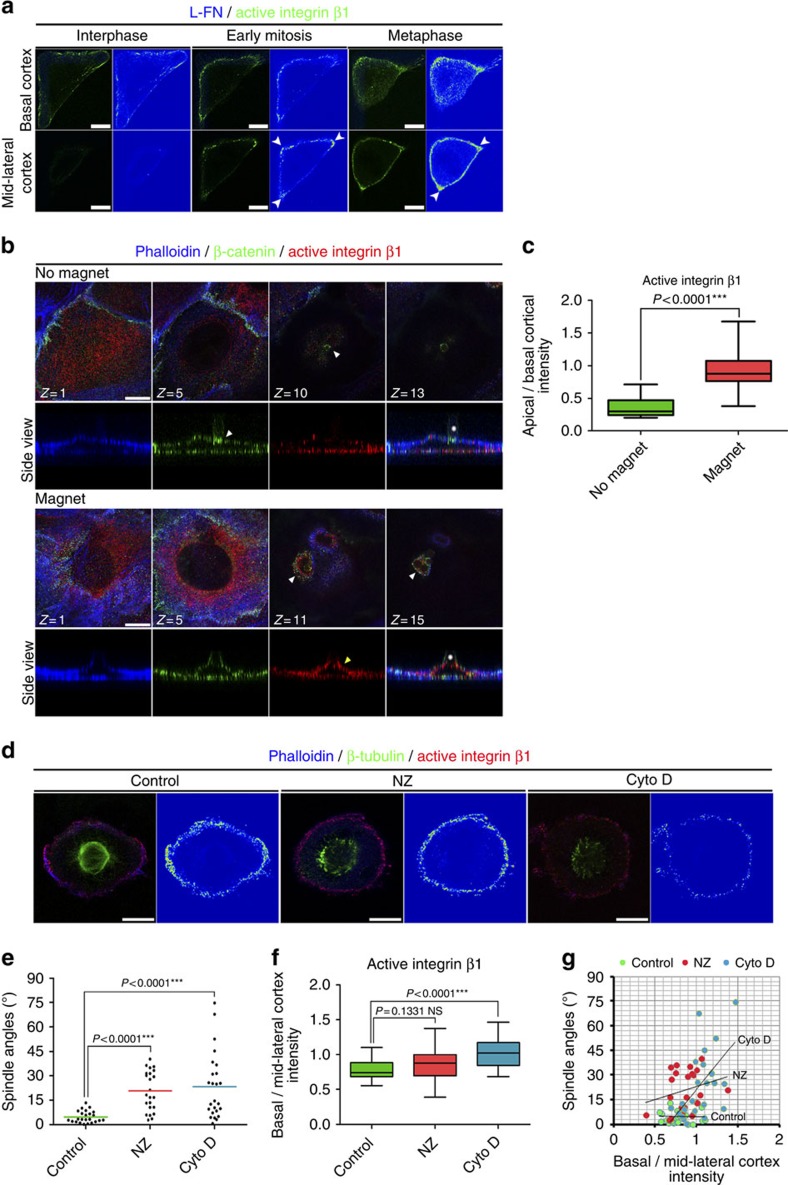
Force-dependent activation of cortical integrin β1. (**a**) Optical sections and colour intensity-coded images at the cell–ECM interface and mid-lateral cortex of interphase, early mitotic and metaphase cells seeded on L-FN microprints. Arrowheads indicate areas where maximal forces are exerted. (**b**) Z-stacks and side view of MCF10A cells with attached E-cadherin-coated beads (asterisk) on their apical surface in the absence or presence of a magnet, imaged under the same conditions. The white arrowhead shows AJs formation between the apical surface and the bead. The yellow arrowhead shows β1 activation at apical surface on force application. (**c**) Box-plot of the apical to basal cortex intensity ratio of active β1 in the presence or absence of force. Mean±s.e.m.: no magnet 0.3745±0.03963, *n*=17; magnet 0.9211±0.06990, *n*=20; *P* values calculated by *t*-test; *n*, number of cells, three independent experiments (one-way analysis of variance: no magnet *P*=0.8469, ns, magnet *P*=0.5004, ns). (**d**) Optical sections at the plane of the spindle and colour intensity-coded image of control, NZ or Cyto D-treated cells, imaged under the same conditions. (**e**) Scatter plot of substrate to spindle angles of the cells in **d**. Mean±s.e.m.: control 4.696±0.7338°, *n*=24; NZ 20.60±2.527°, *n*=23; Cyto D 23.20±4.037°, *n*=25; *P* values calculated by Mann–Whitney test; *n*, number of metaphase cells, two independent experiments (Kruskal–Wallis: Control *P*=0.1615, ns, NZ *P*=0.6323, ns, Cyto D *P*=0.4075, ns). (**f**) Box-plot of the basal to mid-lateral cortex intensity ratio of active integrin β1 of the cells analysed in **e**. Mean±s.e.m.: control 0.7807±0.03210, *n*=24; NZ 0.8597±0.04068, *n*=23; Cyto D 1.030±0.03908, *n*=25; *P* values calculated by *t*-test; *n*, number of metaphase cells, two independent experiments (one-way analysis of variance: control *P*=0.2332, ns, NZ *P*=0.1480, ns, Cyto D *P*=0.7993, ns). (**g**) Correlation of the degree of spindle misorientation with the ratio of basal to mid-lateral cortex intensity of active β1. Pearson's correlation coefficients: control *r*=−0.0289, weak correlation, NZ *r*=0.2547 weak correlation, Cyto D *r*=0.5919 moderate positive correlation. Scale bar, 10 μm (**a**,**b**,**d**).

Evidence has emerged that integrin β1 can be activated through membrane tension suggesting that the activation at the lateral regions of mitotic cells, which are not in contact with the ECM, may be elicited through RF-derived forces^4^,^23^. To test this possibility we stained active β1 in cells adhering on L-fibronectin (FN) microprints, where the areas of the cell receiving maximal force are predetermined^30^,^31^. While in interphase, active β1 is found exclusively on FAs at the interface between the L-FN and the cell, with undetectable lateral cortical staining, during mitosis active β1 becomes enriched at the areas of the cortex that receive the strongest forces (Fig. 3a, white arrowheads) suggesting that force distribution determines the distribution of active β1.

We went on to directly address if integrin β1 can be activated in ECM-free areas in response to force. Paramagnetic beads coated with E-cadherin-Fc were seeded on E-cadherin-expressing cells, allowed to form adherens junctions (AJs) in the absence of serum and were then pulled with a magnet. As shown in Fig. 3b, AJs are formed between the cell and the bead but no active integrin β1 can be detected around the bead. However, on force application, integrin β1 becomes activated on the entire apical cell surface demonstrating that external force is sufficient to activate integrin β1 in the absence of a ligand (Fig. 3b,c). This suggests that the observed β1 activation at the cortex of mitotic cells is force driven.

However, the correlation of active β1 with the spindle capture sites suggests that these forces could be extrinsic through the RFs or intrinsic through the pulling of astral MTs. To discriminate between these two possibilities, we treated cells either with low-dose nocodazole (NZ) to depolymerize astral MTs^32^ or with cytochalasin D (Cyto D) to disrupt the RFs. Although NZ treatment led to spindle misorientation, there was no effect on β1 activation (Fig. 3d–f) confirmed by the lack of a correlation between the degree of spindle misorientation and the ratio of basal-to-lateral active β1 cortical intensity (Fig. 3g). Moreover, NZ treatment of cells on L-FN had no effect on the asymmetric distribution of active β1 (Supplementary Fig. 3a). These results preclude the possibility that integrin β1 activation at the sites of spindle capture is a consequence of the pulling forces from astral MTs. In contrast, Cyto D treatment led to spindle misorientation and nearly eliminated β1 activation on the cortex (Fig. 3d–f, Supplementary Fig. 3a). Importantly, the degree of spindle misorientation was positively correlated with the change of cortex intensity of active β1 linking cortical β1 activation to spindle orientation (Fig. 3g). Cyto D treatment, however, had a visible effect on cortical actin. To ensure that loss of active β1 is not an indirect effect of the disruption of the actin network, we seeded cells on poly-L-lysine (PLL), a substrate on which cells fail to form FAs and RFs^33^. Cells seeded on PLL displayed spindle misorientation and lack of cortical β1 activation, confirming that activation of β1 on the lateral cortex during mitosis depends on RFs^12^ (Supplementary Fig. 3b–d). Overall, these data suggest that integrin β1 can become activated in response to external force and that during mitosis integrin β1 activation depends on RFs.

### Cortically polarized active β1 guides spindle orientation

**Figure 4:**
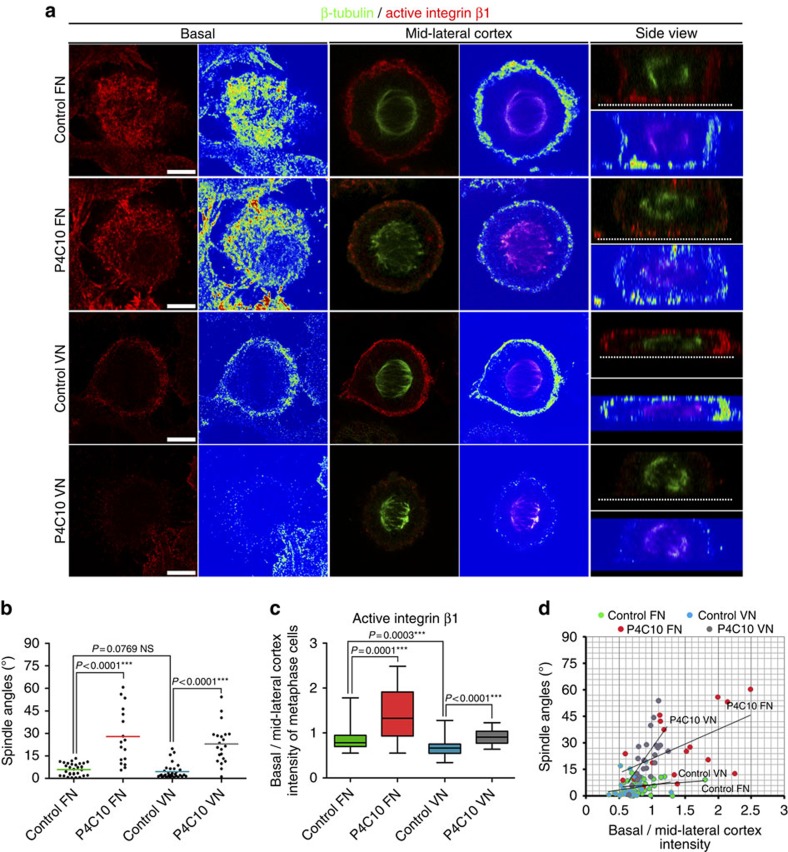
Inhibition of integrin β1 cortical activation leads to spindle misorientation. (**a**) Optical sections at the cell-ECM interface and at the spindle plane, colour intensity-coded images and side views of representative metaphase control or P4C10 antibody-treated HeLa cells seeded on FN or VN. All cells were imaged under the same conditions. Cells were stained with β-tubulin and 9EG7 antibodies. The dashed lines show the cell–ECM interface. (**b**) Scatter plot of substrate to spindle angles of metaphase cells from the above conditions. Mean±s.e.m.: Control FN 5.920±0.7391°, *n*=30; P4C10 FN 27.76±4.455°, *n*=17; Control VN 4.427±0.8606°, *n*=33; P4C10 VN 22.84±2.824°, *n*=21; *P* values calculated by Mann–Whitney test; *n*, number of metaphase cells, two independent experiments (Kruskal–Wallis: control FN *P*=0.3414, ns, P4C10 FN *P*=0.2055, ns, control VN *P*=0.1060, ns, control VN *P*=0.6050, ns). (**c**) Box-plot of the basal to mid-lateral cortex intensity ratio of active integrin β1 of the cells analysed in (**b**). Mean±s.e.m.: control FN 0.8482±0.04543, *n*=30; P4C10 FN 1.388±0.1422, *n*=17; control VN 0.6639±0.02926, *n*=33; P4C10 VN 0.9175±0.03423, *n*=21; *P* values calculated by Mann–Whitney test; *n*, number of metaphase cells, two independent experiments (Kruskal–Wallis: control FN *P*=0.0852, ns, P4C10 FN *P*=0.3592, ns, control VN *P*=0.3051, ns, P4C10 VN *P*=0.5787, ns). (**d**) Correlation of the spindle angle and the ratio of basal to mid-lateral cortex intensity of active integrin β1. Pearson's correlation coefficient: control FN *r*=0.1755 weak correlation, control VN *r*=0.2006 weak correlation, P4C10 FN *r*=0.5601 moderate positive correlation, P4C10 VN *r*=0.645 moderate positive correlation. Scale bar, 5 μm (**a**).

Given the RF-dependent polar activation of integrin β1 at the cortex of mitotic cells and its correlation with spindle orientation we wanted to explore the possibility that this activation has a direct role in spindle orientation. To do so, we had to block ligand unoccupied integrin β1 on the lateral cortex without affecting ligand bound β1 at the cell–ECM interface. We took advantage of the β1-blocking antibody P4C10, which has been shown to preferentially bind unoccupied integrin β1 (refs 34, 35). This antibody would presumably selectively inactivate the cortical pool of β1, which is not ligand bound, whereas engaged integrins at the cell–ECM interface would be unaffected. In agreement with the above, treatment of cells seeded on FN with P4C10, resulted in the reduction of active β1 on the mitotic cortex, while cell adhesion was unaffected (Fig. 4a,c, Supplementary Fig. 4a). Loss of active β1 from the lateral areas of the cortex led to spindle misorientation and the degree of misorientation correlated with the loss of active β1 (Fig. 4b,d). Although these data suggest that activation of β1 on the lateral cortex is necessary for spindle orientation, effects of the antibody on adhesive complexes at the RF–FN interface cannot be ruled out. To address this, we took advantage of cell adhesion to vitronectin (VN). VN interacts with cells through its RGD integrin-binding sequence; however, cell attachment does not depend exclusively on β1 as in the case of adhesion to FN^36^. Moreover, multiple cell types including HeLa cells were shown to also rely on uPAR for adhesion to VN and inhibition of integrin-dependent adhesion on VN does not eliminate cell attachment^37^. As shown, total and active β1 are nearly absent from FAs when cells are seeded on VN (Supplementary Fig. 4b–d). Despite the near undetectable levels of active β1 in interphase cells, cortical activation of β1 in mitotic cells on VN is unaffected and spindles are oriented (Fig. 4a–c). Treatment of cells seeded on VN with P4C10 resulted in loss of cortically active β1 and elicited spindle misorientation despite a lack of any effect on cell adhesion (Fig. 4a–d, Supplementary Fig. 4a). This result confirms that even when cell adhesion is β1-independent, β1 activation on the cortex is necessary for correct spindle orientation.

**Figure 5:**
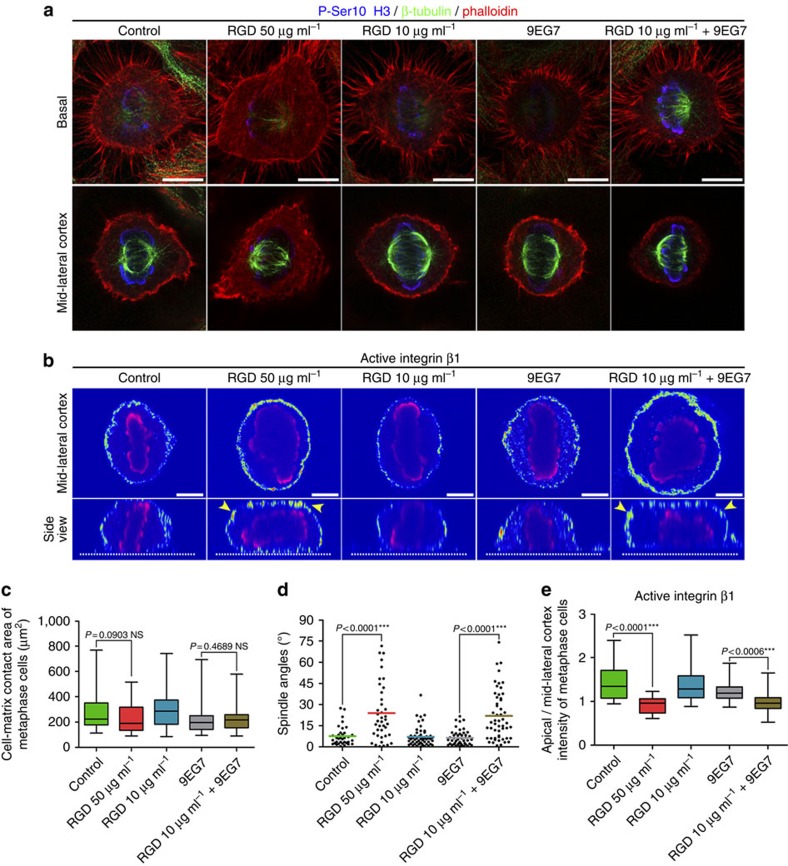
Disruption of the asymmetric distribution of cortically active β1 leads to spindle misorientation. (**a**) Optical sections at the RF and spindle planes of control and treated metaphase cells with RGD 50 μg ml^−1^, RGD 10 μg ml^−1^, 9EG7 antibody, RGD 10 μg ml^−1^+9EG7 antibody. (**b**) Colour intensity-coded optical sections at the spindle plane and side views from metaphase cells under the conditions in **a**. All cells were imaged under the same conditions. The dashed lines show the cell–ECM interface. Yellow arrowheads indicate β1 activation throughout the cortex. (**c**) Box-plot of the cell–matrix contact area. Mean±s.e.m.: control 278.4±24.09 μm^2^, *n*=35; RGD 50 μg ml^−1^ 235.0±19.48 μm^2^, *n*=40; RGD 10 μg ml^−1^ 298.6±20.95 μm^2^, *n*=54; 9EG7 225.1±18.42 μm^2^, *n*=48; RGD 10 μg ml^−1^+9EG7 216.5±11.43 μm^2^, *n*=53; *P* values calculated by Mann–Whitney test; *n*, number of metaphase cells, three independent experiments (Kruskal–Wallis: control *P*=0.0997, ns, RGD 50 μg ml^−1^ *P*=0.1880, ns, RGD 10 μg ml^−1^ *P*=0.7515, ns, 9EG7 *P*=0.1049, ns, RGD 10 μg ml^−1^+9EG7 *P*=0.0557, ns). (**d**) Scatter plot of substrate to spindle angles of cells analysed in **c**. Mean±s.e.m.: control 7.607±1.163°, *n*=35; RGD 50 μg ml^−1^ 23.91±3.219°, *n*=40; RGD 10 μg ml^−1^ 7.100±0.9431°, *n*=54; 9EG7 6.385±0.7696°, *n*=48; RGD 10 μg ml^−1^+9EG7 21.97±2.408°, *n*=53; *P* values calculated by Mann–Whitney test; *n*, number of metaphase cells, three independent experiments (Kruskal–Wallis: control 0.5484, ns, RGD 50 μg ml^−1^ *P*=0.5495, ns, RGD 10 μg ml^−1^ *P*=0.6883, ns, 9EG7 *P*=0.5275, ns, RGD 10 μg ml^−1^+9EG7 *P*=0.8523, ns). (**e**) Box-plot of the apical to mid-lateral cortex intensity ratio of the cells in **b**. Mean±s.e.m.: Control 1.438±0.09659, *n*=21; RGD 50 μg ml^−1^ 0.9443±0.04263, *n*=19; RGD 10 μg ml^−1^ 1.393±0.08771, *n*=20; 9EG7 1.222±0.04880, *n*=20; RGD 10 μg ml^−1^+9EG7 0.9646±0.05918, *n*=19; *P* values calculated by Mann–Whitney test; *n*, number of metaphase cells, two independent experiments (Kruskal–Wallis: Control *P*=0.9148, ns, RGD 50 μg ml^−1^ *P*=0.0777, ns, RGD 10 μg ml^−1^ *P*=0.5638, ns, 9EG7 *P*=0.4708, RGD 10 μg ml^−1^+9EG7 *P*=0.3762, ns). Scale bars 10 μm (**a**), 5 μm (**b**).

Given the importance of integrin β1 cortical activation in spindle orientation, we then asked whether the asymmetric distribution of active β1 is important. We postulated that if β1 has a role in determining the capture sites of astral MTs, then integrin β1 overactivation would lead to spindle misorientation. Treatment of cells with RGD peptide at concentrations that disrupt integrin-dependent adhesion via competition for binding to the substrate has been shown to lead to spindle misorientation^12^. We postulated that treatment of cells adhering on FN with low concentrations of RGD would fail to compete with immobilized FN on the cell's basal region and thus not disrupt adhesion, but bind to and activate unoccupied cortically localized integrins. We found that at 50 μg ml^−1^, RGD did not perturb cell adhesion (Supplementary Fig. 5a–c) or RF formation (Fig. 5a,c). Although at this concentration RGD treatment failed to activate β1 in interphase cells, it elicited robust activation throughout the cortex in mitotic cells (Fig. 5b,e, yellow arrowheads), presumably due to the higher overall membrane tension displayed by such cells^38^, leading to spindle misorientation (Fig. 5a,d). This suggests that the polar distribution of active β1 in mitotic cells is required for correct spindle orientation. To preclude the possibility that undetectable effects of RGD on cell adhesion were responsible, a second approach was implemented where very low concentrations of RGD were combined with an activating integrin antibody (9EG7) which stimulates adhesion. Treatment with 9EG7 was not sufficient to induce ectopic integrin β1 activation because the epitope recognized by 9EG7 is exposed when β1 is bound to a ligand^28^. We thus pretreated cells with lower concentrations of RGD (10 μg ml^−1^) to expose the 9EG7 epitope and then incubated cells with 9EG7 so that ‘primed' integrin molecules can be fully activated. Treatment of cells with 10 μg ml^−1^ RGD peptide had no effect on cell adhesion, spindle orientation, RF formation or integrin activation even in mitotic cells. However, in RGD+9EG7-treated cells, β1 was activated throughout the mitotic cortex and spindles were misoriented despite normal RF formation and cell adhesion (Fig. 5a–e, Supplementary Fig. 5a–c).

Overall, the above data show that both the activation and the asymmetric distribution of active β1 on the cortex are required for correct spindle orientation suggesting that the cortical pool of active β1 is directly involved in spindle orientation.

### Ligand-independent β1 activation guides spindle orientation

**Figure 6:**
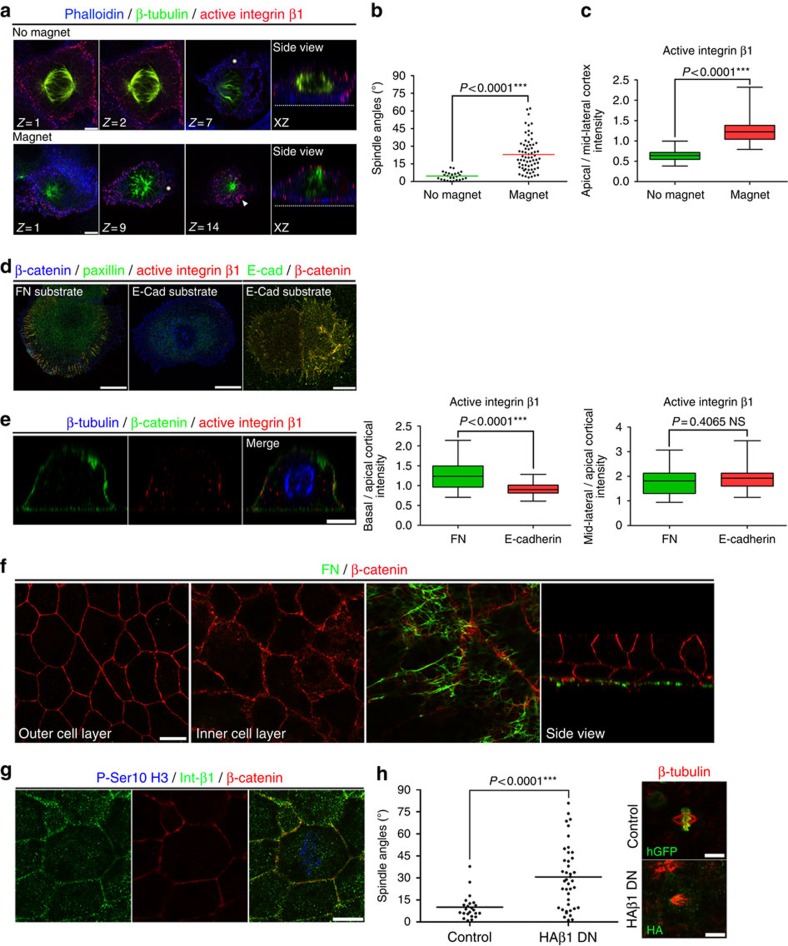
Force-dependent and ligand-independent activation of integrin β1 on the lateral mitotic cortex. (**a**) Optical sections of MCF10A metaphase cells with attached E-cadherin-coated magnetic beads (asterisk) in the presence or absence of a magnet. The white arrowhead indicates β1 activation at the apical surface. (**b**) Scatter plot of substrate to spindle angles for the cells shown in (**a**). Mean±s.e.m.: no magnet 4.604±0.6672°, *n*=25; magnet 22.91±1.745°, *n*=67; number of metaphase cells, three independent experiments (Kruskal–Wallis: no magnet *P*=0.0963, ns, magnet *P*=0.5604, ns) (**c**) Box-plot of the ratio of apical to mid-lateral cortex intensity of active β1 for the cells in **a**. Mean±s.e.m.: no magnet 0.6418±0.02712, *n*=25; magnet 1.256±0.03875, *n*=67; *P* values calculated by Mann–Whitney test; *n*, number of metaphase cells, three independent experiments (Kruskal–Wallis: no magnet *P*=0.5099, ns, magnet *P*=0.7475, ns). (**d**) Confocal images of MCF10A interphase cells seeded on FN or E-cadherin-Fc-coated silanized coverslips. (**e**) Side view of an MCF10A metaphase cell seeded on E-cadherin-Fc and box-plots of basal/apical and mid-lateral/apical cortex intensity ratio of active β1 on FN versus E-cadherin substrate. Mean±s.e.m.: basal/apical: FN 1.260±0.07387, *n*=21; E-cadherin 0.9181±0.03399, *n*=21; mid-lateral/apical: FN 1.800±0.1238, *n*=21; E-cadherin 1.948±0.1149, *n*=21; *P* values calculated by Mann–Whitney test; *n*, number of metaphase cells, three independent experiments (Kruskal–Wallis for basal/apical: FN *P*=0.4231, ns, E-cadherin *P*=0.3451, ns; for mid-lateral/apical: FN *P*=0.7960, ns, E-cadherin *P*=0.754 ns). (**f**) Optical sections at the Xenopus outermost epithelial layer, innermost epithelial layer, basal surface of the inner epithelial layer and a side view. (**g**) Optical section at the mid-lateral cortex of a mitotic cell of the Xenopus outermost epithelium. (**h**) Representative top views of mitotic control cells (histone GFP injected) or cells injected with integrin β1 dominant-negative (HAβ1-DN) of the Xenopus epidermis and a scatter plot of the apical surface to spindle angles of these cells. Mean±s.e.m.: control 10.06±1.758°, *n*=23; HAβ1-DN 30.66±3.389°, *n*=41; *P* value analysed by Mann–Whitney test; *n*, number of metaphase cells, two independent experiments (Kruskal–Wallis: Control *P*=0.7215, ns, HAβ1 *P*=0.92 ns). Scale bar, 5 μm (**a**,**e**), 10 μm (**d**,**f**–**h**).

The above data collectively suggest that integrin β1 becomes activated in the absence of ligand and guides spindle orientation. To address directly whether force application is sufficient to activate integrin β1 on the cell cortex independently of ligand binding and influence spindle orientation, we applied external force on mitotic cells using E-cadherin-coated paramagnetic beads as described in Fig. 3b. Cells were either fixed directly or after placing a magnet above them for 30 min. As shown in Fig. 6a, in the absence of external force the mitotic spindle is oriented properly parallel to the substrate and active β1 is primarily enriched at the mid-lateral cortex. However, on force application integrin β1 becomes activated throughout the cell cortex and the orientation of the mitotic spindle becomes randomized (Fig. 6a–c), showing that application of force perpendicular to the substrate plane leads to ectopic β1 activation on the apical cortex eliciting reorientation of the spindle.

To address directly whether β1 activation at the mid-lateral cortex is ligand independent, we seeded E-cadherin expressing cells in an integrin-independent manner and examined whether β1 becomes activated at the lateral cortex during mitosis. Cells were seeded on E-cadherin-Fc immobilized on silanized glass^39^ in the absence of serum and allowed to attach for 2 h. As previously described^40^,^41^, the cells attached through the generation of AJs on this substrate (Fig. 6d). Under these conditions the cells formed no detectable FAs and no active integrin β1 could be detected in interphase cells, while β-catenin-positive adhesions were free of active β1 (Fig. 6d). However, active β1 was readily detected in mitotic cells at the lateral cortex, and enriched at the spindle capture sites but excluded from the basal surface (Fig. 6e). These results confirm that integrin β1 is activated in a ligand-independent manner in mitotic cells.

Previous work revealed that FAK, a major downstream effector of integrins, is required for correct spindle orientation in the *Xenopus* outermost epithelium, a tissue where cells orient their spindles in an ECM-independent manner^5^,^20^. The outer epithelial cells of *Xenopus* are not in contact with the ECM and do not form FAs or RFs. This tissue consists of two cell layers attached through AJs so the outer layer, as shown (Fig. 6f), is never directly in contact with the ECM^20^,^42^. Nevertheless, integrin β1 is expressed in the outer cells localizing at their basolateral region (Fig. 6g). To address a possible role of β1 in spindle orientation in this tissue we expressed a previously characterized dominant-negative^20^,^43^. As shown, blocking integrin β1 function leads to spindle misorientation suggesting a conserved ligand-independent role of β1 in spindle orientation in embryonic tissues (Fig. 6h).

Taken together, these results suggest that during mitosis integrin β1 regulates spindle orientation in response to external forces in a ligand-independent manner.

### A mechanosensory complex is formed on the mitotic cortex

We went on to explore the mechanism by which β1 activation modulates spindle capture on the cortex. Astral MT capture is achieved through the capture machinery and previous work suggested that cortical accumulation of LGN/NuMA is compromised on loss of the RFs after drug treatments^44^,^45^. Although this was attributed to a role of actin filaments in the recruitment of LGN/NuMA to the cortex it also raised the possibility that β1 activation may influence the cortical recruitment of LGN/NuMA since Cyto D also eliminated β1 activation. To test this possibility, we examined the localization of LGN/NuMA in cells seeded on PLL that display no β1 activation but have an intact actin cytoskeleton. As shown, although cells on PLL display spindle misorientation, the cortical accumulation of LGN and NuMA are unaffected and both are enriched at the regions of the cortex proximal to the spindle poles, marking the spindle capture sites (Supplementary Fig. 6). These results show that the spindle-cortex link is unaffected in the absence of β1 activation and suggest that integrin β1 orients the mitotic spindle though a capture machinery-independent mechanism.

**Figure 7:**
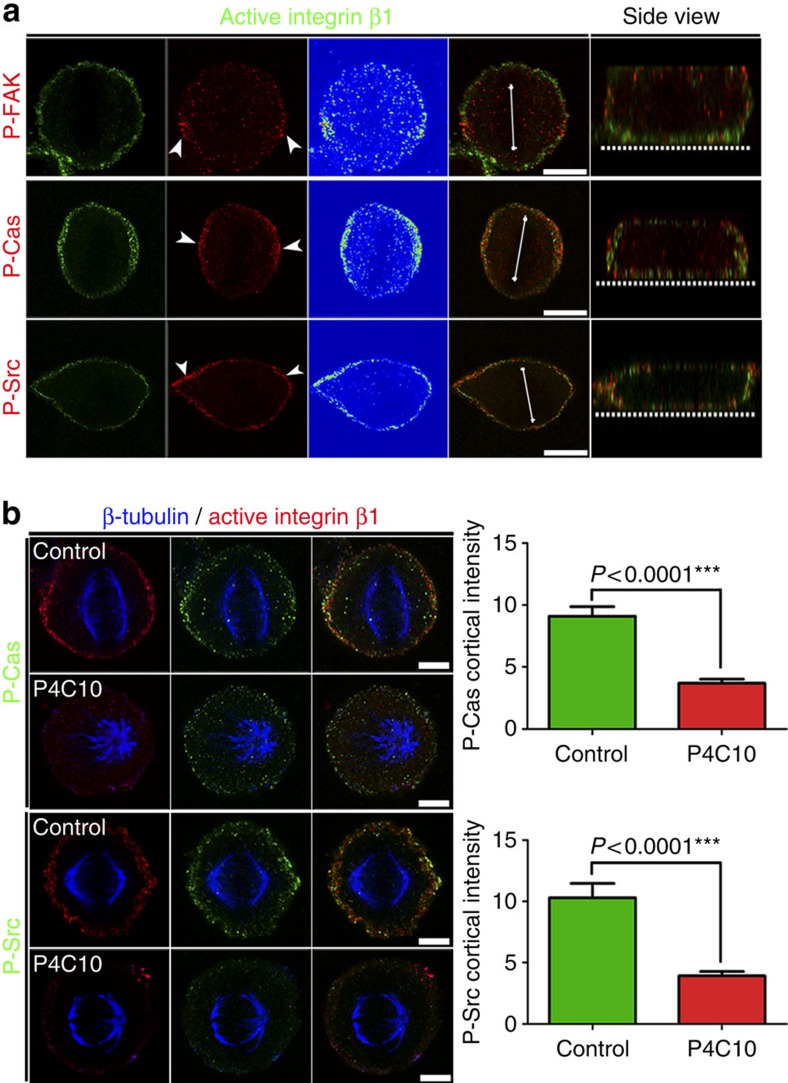
An integrin β1-based cortical mechanosensory complex is formed at the lateral cortex of mitotic cells. (**a**) Optical sections, colour intensity-coded images and side views of representative metaphase HeLa cells co-stained for active β1 and phosphorylated active forms of FAK, Cas or Src. The white arrowheads indicate the polarized cortical crescent of the phosphorylated forms of the above proteins. The white line represents the metaphase plate and the dashed line shows the basal surface. (**b**) Optical sections at the spindle plane of metaphase control HeLa cells or cells treated with the P4C10 antibody. All cells were imaged under the same conditions. Cells were co-stained for β-tubulin, active β1 (9EG7) and phosphorylated Cas or Src. The plots show the average cortical intensity of P-Cas and P-Src in control and P4C10-treated cells. mean±s.e.m.: P-Cas control 9.106±0.7618, *n*=33; P-Cas P4C10 3.691±0.3323, *n*=34; P-Src control 10.30±1.155, *n*=35; P-Src P4C10 3.916±0.3537, *n*=30. Analysed by Mann–Whitney test; n, number of metaphase cells, two independent experiments (Kruskal–Wallis: for P-Cas Control *P*=0.0715, ns, P4C10 *P*=0.2177, ns; for P-Src Control *P*=0.0657, ns, P4C10 *P*=0.2510, ns). Scale bar, 10 μm (**a**), 5 μm (**b**).

Work by the Humphries group has shown that β1 activation at FAs leads to targeting of MTs at the cortex and their stabilization at this region^46^. This, coupled with our previous findings concerning the role of FAK in spindle responses to external force^5^, raised the possibility that FA-like complexes form in response to β1 activation at the cortex preferentially guiding and stabilizing astral MTs towards regions receiving external forces. We began by examining the localization of phosphorylated FAK. Active FAK is localized at FAs in interphase cells^47^, however, during mitosis it is detected on the cell cortex (Fig. 7a). Given previous data showing that ligand-independent activation of integrin β1 elicits Cas phosphorylation^23^ and its direct interaction with FAK^48^,^49^, we also examined the localization of phosphorylated Cas. As shown, P-Cas also becomes localized at the mitotic cortex and the same is true for Src, the principle kinase shown to phosphorylate Cas (Fig. 7a) (refs 50, 51). In addition, the active forms of all three proteins display lateral cortical polarity in a similar manner to active β1 (Fig. 7a, white arrowheads). Importantly, loss of β1 cortical activation through P4C10 treatment led to a reduction of P-Cas and P-Src cortical localization suggesting that the localization/phosphorylation of these proteins on the cortex of mitotic cells depends on β1 activation (Fig. 7b). These results suggest that a cortical mechanosensory complex (CMC) forms at the lateral regions of the cortex in non-polar adherent cells and asked if this complex also forms in polarized cells. Staining of MDCK monolayers revealed that active β1, P-FAK, P-Cas and P-Src are localized at the lateral aspect of the cortex specifically in mitotic cells in a similar manner to HeLa cells (Supplementary Fig. 7a,b). Furthermore, examining the localization of P-FAK, P-Cas and P-Src in the outermost epithelium of *Xenopus* embryos reveals that although all cells of the epithelium display staining on the lateral regions of the cortex (especially multiciliated cells), mitotic epithelial cells display a clear elevation of this signal (Supplementary Fig. 7c, white arrowheads). Overall, the above data suggest that ligand-independent β1 activation leads to the formation of conserved cortical complex in mitotic cells irrespective of cell or tissue context.

### The members of the CMC cooperate to orient the spindle

**Figure 8:**
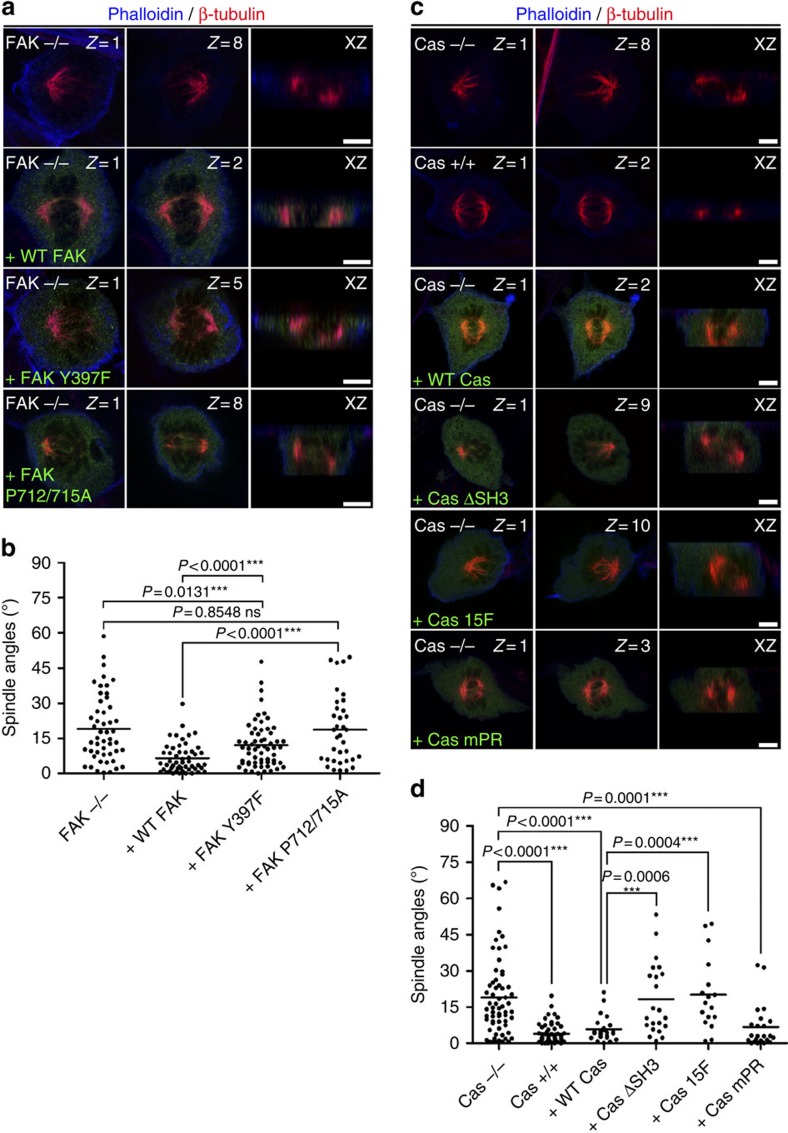
Interactions between the members of the CMC guide mitotic spindle orientation. (**a**) Optical sections at the plane of each spindle pole and side views of FAK nulls, or FAK nulls transfected with the indicated constructs (signal shown in green) stained for β-tubulin and actin. (**b**) Scatter plot of the substrate to spindle angles of metaphases cells described in **a**. Mean±s.e.m.: FAK−/− 19.04±2.028°, *n*=50; FAK−/− +WT FAK 6.507±0.8685°, *n*=52; FAK−/− +FAK Y397F 12.14±1.263°, *n*=61; FAK−/− +FAK P712/715 A 18.63±2.501°, *n*=35; *P* values calculated by Mann–Whitney test; *n*, number of metaphase cells, two independent experiments (Kruskal–Wallis: FAK−/− *P*=0.5405, ns, +WT FAK *P*=0.2432, ns, +FAK Y397F *P*=0.1732, ns, +FAK P712/715 A *P*=0.0.8886, ns). (**c**) Optical sections at the plane of each spindle pole and side views of Cas nulls, Cas reconstituted cells or Cas nulls transfected with the indicated constructs (shown in green) stained for β-tubulin and actin. (**d**) Scatter plot of the substrate to spindle angles of metaphases cells described in **c**. Mean±s.e.m.: Cas−/− 18.97±2.102°, *n*=63; Cas+/+ 3.825±0.5526°, *n*=57; Cas−/−+WT Cas 5.805±1.062°, *n*=24; Cas−/−+Cas ΔSH3 18.24±3.173°, *n*=22; Cas−/−+Cas 15 F 20.09±3.660°, *n*=17; Cas−/−+Cas mPR 6.703±1.882°, *n*=23; *P* values calculated by Mann–Whitney test; *n*, number of metaphase cells, two independent experiments (Kruskal–Wallis: Cas−/− *P*=0.6765, ns, Cas+/+ *P*=0.1978, ns, +WT Cas *P*=3967, ns, +Cas ΔSH3 *P*=0.7745, ns, +Cas 15 F *P*=0.2913, ns, +Cas mPR *P*=0.1564, ns). Scale bar, 5 μm (**a**,**c**).

Our data suggest that a complex composed of FAK, Cas and Src forms on active β1 at the lateral cortex of mitotic cells and regulates spindle responses to external forces. We went on to explore the interactions between its members that are important in this process. Expression of a Cas-binding defective mutant of FAK (FAK P712/715A)^48^ failed to rescue spindle orientation defects in both FAK null mouse embryonic fibroblasts derived from FAK KO mice^52^ and in epithelial cells of FAK morphant *Xenopus* embryos, suggesting that the FAK-Cas interaction is important (Fig. 8a,b, Supplementary Fig. 8a). Since Cas had not been previously implicated in this process we examined Cas null fibroblasts derived from Cas knockout mice^53^ that, as shown, display spindle misorientation unlike Cas reconstituted cells (Fig. 8c,d). In addition, expression of the CasΔSH3 mutant, which does not bind FAK, fails to rescue defects of Cas null cells confirming the importance of this interaction in spindle orientation^48^ (Fig. 8c,d). Given the previously characterized role of the substrate domain (SD) of Cas in mechanotransduction from FAs^54^, we asked if phosphorylation of Cas is important. As shown, expression of the Cas 15F mutant failed to rescue spindle misorientation (Fig. 8c,d). The SD domain of Cas is a well-characterized substrate of Src kinases^50^,^51^ and it has been previously shown that the Src kinase activity is indispensable for force-dependent spindle orientation^31^. This raised the possibility that during mitosis Cas is phosphorylated by Src. SD phosphorylation is mediated through two distinct mechanisms; either through direct binding of the SH3 domain of Src to the C-terminal polyproline region of Cas or via indirect association of Src with Cas through FAK^55^. As shown, expression of the Cas mPR mutant in which the Src-Cas interaction is impaired rescues as effectively as WT Cas^56^,^57^,^58^ (Fig. 8c,d), suggesting that direct binding of Src on Cas is dispensable. Expression of a Src binding mutant of FAK (Y397F) in FAK nulls, however, was significantly less effective in rescuing spindle orientation defects compared with WT FAK, suggesting that Src binding on FAK is important (Fig. 8a,b). The requirement of Y397 and of Src's kinase activity to phosphorylate Cas in the orientation of the mitotic spindle were confirmed *in vivo*, where use of a Src inhibitor also blocked Cas phosphorylation, providing further support for the notion that the β1-dependent CMC is conserved in the embryo (Supplementary Fig. 8b,c). Overall, these data show that direct interaction of FAK with Cas and Src is critical for correct spindle orientation and suggest that during mitosis a complex forms downstream of force-dependent integrin β1 activation in which FAK acts as a scaffold to elicit Src-dependent Cas phosphorylation.

## Discussion

The involvement of integrins in spindle orientation has been established for almost a decade now. Integrin β1 knockout mice display orientation defects in asymmetric divisions of basal keratinocytes during stratification^13^, while disruption of β1-dependent cell adhesion leads to spindle misorientation with respect to the substrate^12^. Studies have implicated integrins in the regulation of divisions in polarized epithelial cells^15^,^17^. However, a direct role in spindle orientation in this context is difficult to conclude given the fact that integrins regulate basement membrane deposition and apicobasal polarity, raising the possibility that the observed spindle orientation defects may be secondary to defects in epithelial polarity^16^. Overall, intergins are broadly implicated in the regulation of spindle orientation through their interactions with ECM components and signalling from adhesion complexes suggesting that their role in spindle orientation stems from their role in cell adhesion.

In this study, we show that integrin β1 is involved in spindle orientation independently from its role in cell adhesion and provide insight into the molecular link between spindle orientation and integrins. We show that integrin β1 becomes activated on the mitotic cortex in a polarized manner, displaying asymmetry along the apicobasal axis (*z* axis) and at the plane of the spindle (*x*–*y* plane). Specifically, active β1 is enriched at the lateral regions of the cortex where the RFs terminate, while there is no active β1 at the apical regions that are devoid of RFs. The distribution of active β1 at the plane of the spindle is polar correlating with the distribution of RFs and displaying enrichment at the spindle capture sites. We have previously shown that spindle orientation in non-polarized cells along the *z*-axis and the *xy* plane both rely on the three-dimensional distribution of RFs and subsequently the distribution of forces on the cortex^5^. Similarly, the asymmetric activation of integrin β1 in areas that are not in contact with ECM depends on the asymmetry in the distribution of the RFs, suggesting that force transmitted through the RFs activates β1 on the cortex.

The notion that integrin β1 can be activated in a ligand-independent manner was initially proposed by Ferraris *et al.* Through a series of experiments, taking advantage of UPAR's ability to bind VN, the authors showed that integrin β1 can be activated through membrane tension in cells adhering in an integrin-independent manner. In addition, the authors showed that this activation leads to signalling from active β1 (ref. 23). The physiological relevance of this mode of activation and whether it actually occurs in parallel with ligand-dependent activation was not clear, given the artificial nature of the system used. Nevertheless, the authors convincingly demonstrated that integrin β1 can be activated by membrane tension and here we provide evidence that β1 can be activated through direct application of force and that ligand-dependent and -independent activation of integrin β1 co-exist in mitotic cells. Specifically, we show that cells adhering in an integrin β1-independent manner display integrin β1 activation at the lateral mitotic cortex that is independent from β1 activation at the cell–ECM interface. It should be noted that although it was previously suggested that some integrin heterodimers that include the β1 subunit can bind E-cadherin or VN^59^,^60^, under the conditions of these experiments no β1 activation could be detected at the cell–substrate interface.

The activation of integrin β1 on the cortex of mitotic cells in conjunction with previous work implicating it in spindle orientation, suggests that β1 has ligand-dependent and ligand-independent roles in this process. Ligand-dependent roles stem from the involvement of integrins as receptors for cell adhesion and additionally, through the requirement of signalling through β1 for proper polarization of epithelia^16^. Separating the two is not trivial however, our data showing that loss of active β1 from the cortex as well as loss of its asymmetric distribution lead to spindle misorientation, suggest that cortical activation of β1 is a major determinant for correct spindle orientation. Importantly, inhibition of β1 activation in cells that orient in an ECM-independent fashion also leads to spindle misorientation, providing strong support for a ligand-independent role of β1 in this process.

How is β1 activation linked to force sensing by the spindle? Our data show that proteins which normally reside at FAs are recruited to the lateral regions of the cell cortex during mitosis where they co-localize with and display similar asymmetric distributions as active β1. The recruitment of these proteins to the cortex depends on integrin β1, suggesting that β1 activation leads to the formation of a conserved cortical complex responsible for facilitating signal transduction from force activated β1 to the spindle. Disrupting the interactions between its members, results in defective spindle orientation in both adherent cells and embryonic epithelia.

**Figure 9:**
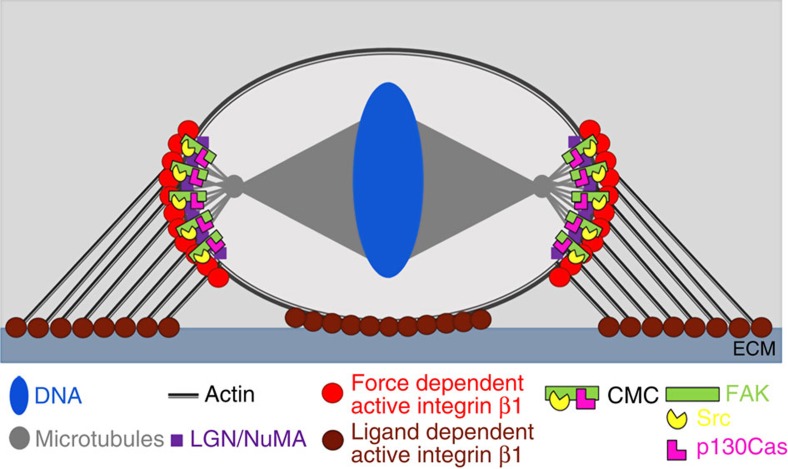
Ligand-independent integrin β1 activation guides spindle orientation. Schematic illustration of the distribution of the two pools of active integrin β1 in mitotic cells: Ligand-dependent active β1 is localized at the cell–ECM interphase whereas the ligand-independent and force-dependent active β1 is enriched at the areas where the RFs terminate on the lateral cortex and specifically enriched at the spindle capture sites. Integrin β1 activation on the lateral cortex results in the establishment of the CMC, which guides spindle capture in response to force.

We propose that in mitotic cells there are two pools of active β1 one at the cell–ECM interface which is ligand and force dependent and one at the mid-lateral regions of the cortex, which is force dependent but ligand independent (Fig. 9). Although the ligand-dependent pool is essential for cell adhesion it is not directly involved in spindle orientation. In contrast, the ligand-independent pool is necessary for correct spindle responses to external force through the establishment of the CMC (Fig. 9). The mechanism through which the CMC influences spindle capture is not clear however, a subcortical actin pool has been suggested to interact with myosin 10 at the astral MTs^61^ and to influence their capture sites on the cortex^4^. Phosphorylation of Cas initiates Rac activation^62^,^63^ and it is possible that this actin pool is regulated through localized activation of Rac. In agreement with this, Rac activity is polarized and regulates anchoring of the spindle in mammalian oocytes^64^. The possibility that cortical β1 activation guides astral MTs is supported by studies showing that MTs target sites of integrin activation and the presence of active integrins stabilizes MTs at the cell cortex^46^,^65^. Since cortical activation of β1 depends on RF-derived forces and the greatest forces are exerted at the mid-lateral region where the RFs are longer, astral MTs would presumably be stabilized preferentially at these areas, promoting capture at the lateral rather than the basal cortex explaining why the spindle is oriented parallel to the substrate. In addition, the exclusion of a vertical division can also be explained by the absence of the capture machinery at the apical cortex of the cells where no force is applied. However, since our data suggest that β1 activation does not directly influence the recruitment of LGN/NuMA to the cortex, it is likely that the exclusion of LGN/NuMA from the apicobasal cortex in metaphase cells is secondary to lateral capture and a result of the chromosome-derived RanGTP gradient shown to restrict the localization of NuMA-LGN^66^.

Overall, our work reveals a conserved ligand-independent role of integrin β1 in spindle orientation and suggests that a CMC composed of well-characterized FA proteins is responsible for spindle responses to external force.

## Methods

### Cell culture and transfections

HeLa and MDCK cells (ATCC) were cultured in DMEM with 10% FBS. FAK null cells (ATCC) were cultured in DMEM with 10% FBS and 1 mM sodium pyruvate. Cas null and Cas reconstituted cells (provided by Dr Sara Cabodi) were cultured in DMEM with 10% FBS and 1 mML-Glutamine. MCF10A cells (ATCC) were cultured in DMEM-F12 supplemented with 5% horse serum, 20 ng ml^−1^ EGF, 0.5 mg ml^−1^ hydrocortisone, 100 ng ml^−1^ Cholera Toxin and 10 μg ml^−1^ Insulin. Transfections in all cell lines were performed with Lipofectamin 2,000 (11668019, Invitrogen) according to the manufacturer's instructions.

### Cell adhesion on substrates

Coating of charged coverslips with FN and VN was performed for 2 h at 37 °C prior cell seeding. PLL coating was performed for 30 min at room temperature (RT). In all experiments except those where parallel cell adhesion on FN, VN or PLL was performed, cells were adhered overnight on 10 μg ml^−1^ FN (Invitrogen) coated coverslips. For experiments comparing cells seeded on FN versus VN, cells were seeded on 10 μg ml^−1^ FN or 20 μg ml^−1^ VN (Sigma) for 4 h before fixation to preclude FN deposition and fibrillogenesis on VN, which was verified via FN immunostaining. Cells on PLL were adhered for 1 h before fixation. MCF10A cells were seeded on silanized coverslips coated with mouse E-cadherin-Fc chimera (748-EC-050, R&D Systems). Silanization of charged coverslips was performed according to established protocols^39^. Briefly, coverslips were hydroxylated in saturated KOH in isopropanol, overnight at RT, washed three times with water and dried. Then coverslips were treated with 1% (3-aminopropyl)trimethoxysilane (281778, Sigma) in toluene, overnight at RT, washed three times in toluene and dried. E-cadherin coating was performed at 3 μg ml^−1^ for 2 h at 37 °C and crosslinked. MCF10A cells were mechanically detached in PBS containing 10 mM EDTA and 2% BSA and seeded in DMEM-F12 media for 90 min before fixation. Plating of cells on FN micropatterned coverslips (CYTOO) was performed according to the manufacturer's protocol.

### Magnetic beads

A mixture of 1.5 μg of E-cadherin-Fc and 2 mg ml^−1^ of Protein A dynabeads (100.02D, Invitrogen) was incubated for 20 min at RT with intermediate slight vortex steps. Beads were recovered with the use of a magnet, washed twice in PBS, resuspended in 30 μl of DMEM-F12 and added for 2 h in the absence of serum on MCF10A seeded cells on coverslips. Cells were then either fixed directly or after the beads were pulled for 15 min by placing a magnet at the edge of the coverslip. For addressing the effects of application of external force on spindle orientation, cells with attached beads were either fixed directly or after the beads were pulled for 30 min by placing the magnet above them.

### DNA Constructs and morpholinos

All plasmids generated were verified by sequencing.

All FAK mutants were generated from the FAK chicken variant (GenBank AAA48765.1). The constructs WT FAK, FAK K454R and FAK Y397F in pCS2++ vector have been described (citations). The hemaglutinin (HA) or GFP-tagged version of the FAK P712/715A construct was generated by site-directed mutagenesis using the set of primers F/FAK P712/715A: 5′-GGATCAGATGAAGCTGCTCCCAAGGCCAGCAGGCCTGGTTAC-3′ and R/FAK P712/715A: 5′-GTAACCAGGCCTGCTGGCCTTGGGAGCAGCTTCATCTGATCC-3′. The plasmid histone GFP pCS2+ was kindly provided by Chenbei Chang lab. The pEGFP C1-LGN construct was kindly provided by Fumiko Toyoshima Lab. The HAβ1 pSP64T construct was provided by Douglas DeSimone lab. The pCS2 GFP-Utrophin plasmid was provided by Dr John Wallingford. All p130Cas constructs were provided by Dr Steven Hanks. The sequence of FAK morpholino (MO) is 5′-TTGGGTCCAGGTAAGCCGCAGCCAT-3′.

### Immunostaining

Immunofluorescence on HeLa, MDCKs, FAK null and Cas null cells was carried out as follows: cells were fixed for 15 min in 4% paraformaldehyde. When staining phosphorylated proteins, fixation was performed in the presence of 1 mM Sodium Orthovanadate. Fixation was followed by addition of 50 mM glycine. When active or total integrin β1 staining was performed, cells were first blocked in 10% normal donkey serum (Jackson Immunoresearch) and incubated with the integrin antibodies before triton treatment to preserve the specificity of the antibodies against their epitopes for 90 min at RT. Cells were then washed, permeabilized using 0.03% Triton-X for 6 min, blocked again for 15 min and incubated with the primary antibodies for 90 min RT. The primary antibodies used are: active integrin β1 9EG7 (1:250, 550531 BD Pharmigen), active integrin β1 HUTS-21 (1:250, 556048, BD Pharmigen), integrin β1 K20 (1:50, sc-18887, Santa Cruz), integrin β1 TS2/16 (1:500, sc-53711, Santa Cruz), integrin β1 AIIB2 (1:500, Hybridoma Bank), β-tubulin (1:200, E7, Hybridoma Bank), FAK (1:500, 05–537, Millipore), Paxillin (1:1,000, 610569, BD Biosciences), P-Ser10 H3 (1:1,000, 06–570, Millipore), P-Y576 FAK (1:200, 700013, Invitrogen), P-Y165 CAS (1:50, 4015, Cell Signaling), P-Y416 Src (1:100, 2101, Cell Signaling), β-catenin (1:500, sc-7199, Santa Cruz), NuMA (1:500, ab36999, Abcam). Cells were then washed several times in PBS and incubated with secondary antibodies. Staining of the f-actin was performed using phalloidin (Invitrogen), whereas DNA staining was performed by the use of TO-PRO 3 (Invitrogen). Mounting was performed in prolong gold antifade media.

Embryos were fixed in 10% 10XMEMFA, 10% 3.7% formaldehyde and 80% water for 2 h at RT, permeabilized in PBST (1 × PBS, 0.5% Triton, 1% dimethyl sulfoxide) and blocked for 30 min in 10% Normal Donkey serum. Primary antibodies were incubated overnight at 4 °C. The primary antibodies used are: β-tubulin (1:200, E7, Hybridoma Bank), GFP (1:500, A11122, Invitrogen), HA (1:250, sc-805, Santa Cruz), β-catenin (1:500, sc-7199, Santa Cruz), FN (1:200, 4H2, provided Dr Douglas DeSimone), integrin β1 (1:50, 8C8, Hybridoma Bank). Embryos were washed in PBST and incubated for 2 h with secondary antibodies at RT, washed several times and post-fixed in 1XMEMFA. Clearing of the embryos was performed after methanol dehydration followed by immersing them in Murray's Clearing Medium.

### Live cell staining

Live imagining of active integrin β1 was performed by treating seeded cells for 30 min with the 9EG7 antibody (0.15 μg ml^−1^) in media, followed by three media washes and incubation with Cy-3 anti-rat secondary antibody for 30 min. Cells were then washed with media and imaged.

### Drugs, peptides and antibodies treatments

HeLa cells were treated with 10 nM of NZ for 30 min and 0.5 μg ml^−1^ of Cyto D for 1 h at 37 °C. For integrin blocking experiments, the P4C10 antibody (antibody supernatant from Hybridoma Bank, 1:100 dilution) was added to the cells for 30 min at 37 °C before fixation. For integrin overactivation experiments, cells were treated with 10 μg ml^−1^ or 50 μg ml^−1^ of RGD peptide for 1 h, or with 0.625 μg ml^−1^ 9EG7 antibody for 1 h or with 10 μg ml^−1^ of RGD for 30 min followed by treatment with 10 μg ml^−1^ of RGD and 0.625 μg ml^−1^ 9EG7 for another 30 min at 37 °C. *Xenopus* embryos were treated with 8 μΜ of Src inhibitor PP2 (Sigma) from stage 9 until they were fixed at stage 15.

### Embryos and manipulations

*Xenopus* laevis embryos were staged according to Nieuwkoop and Faber (1967). Embryos were fertilized *in vitro* and dejellied using 1.8% L-cysteine, pH 7.8, then maintained in 0.1 × Marc's Modified Ringer's. Microinjections were performed in 4% Ficoll in 0.3 × Marc's Modified Ringer's. For spindle orientation in the *Xenopus* outermost epidermis embryos were injected with 50 ng of FAK MO at the one cell stage. All FAK constructs were co-injected with the FAK MO at 300 pg. Embryos were allowed to develop until stage 9, where animal caps were dissected and cultured in Danilchik's for Amy (DFA; 53 mM NaCl_2_, 5 mM Na_2_CO_3_, 4.5 mM potassium gluconate, 32 mM sodium gluconate, 1 mM CaCl_2_, 1 mM MgSO_4_) until stage 15, when they were fixed and processed for immunofluorescence. HAβ1 construct was injected at the one cell stage at 300 pg.

### Imaging

Cells and embryos were imaged on a laser scanning confocal microscope (LSM710 Zeiss).

### Statistical Analysis

For all independent experiments, at least two replicates were performed for each experiment (defining as replicate each coverslip with attached cells). The data obtained from the replicates were pulled together and analysed either with *t*-test for parametric distributions or with Mann–Whitney test for non-parametric distributions as judged by the D'Agostino–Pearson normality test. When noted in the figure legends, data were also analysed with one-way analysis of variance or Kruskal–Wallis tests to estimate the variation within each experimental group between the replicates.

Quantification of the orientation of the division axis of metaphase cultured and epithelial cells was carried out with ImageJ using the Z-projections of the images. The angle between the line connecting the two spindle poles and the line extending from the one spindle pole parallel to the substrate was measured. Quantification of spindle orientation in the cells of the outermost cell layer of the *Xenopus* epithelium was carried out as described above, by measuring the angle between the line connecting the two spindle poles and the line extending from the one spindle pole parallel to the apical surface of the cell. Statistical analysis of spindle orientation including two-tailed unpaired *t*-test for parametric distributions and graph construction were performed by the GraphPad software.

Intensities and cell-matrix contact areas were measured using the Zen 2010 software. Intensity ratios were calculated in Microsoft Excel 2013 and all measurements were analysed with GraphPad.

The correlation of spindle orientation to the active integrin β1 cortical polarity crescent was performed by measuring the angle formed between the line connecting the centre of the metaphase plate perpendicularly to the cell cortex and the line extending from the centre of the active integrin β1 cortical crescent towards the centre of the metaphase plate. The angular distribution plot was constructed by using MATLAB.

The correlation of spindle misorientation with the levels of integrin β1 cortical activation was performed by measuring the spindle angle and the ratio of basal to mid-lateral cortex intensity of active integrin β1 for each metaphase cell. Pearson correlation coefficient (*r*) was calculated for the two variances, where −1<*r*<1 and graphs were constructed in Microsoft Excel 2013.

The correlation of LGN cortical crescent with spindle orientation was performed by measuring the angle α (Supplementary Fig. 6a) formed between the line connecting the centre of the metaphase plate perpendicularly to the cell cortex and the line extending from the centre of LGN cortical crescent towards the centre of the metaphase plate. The correlation of NuMA cortical centre with spindle orientation was performed by measuring the angle β (Supplementary Fig. 6d) formed between the extending line connecting the two spindle poles towards the cell cortex and the line extending from the centre of NuMA cortical crescent towards the proximal spindle pole.

## Additional information

**How to cite this article:** Petridou, N. I. *et al.* A ligand-independent integrin β1 mechanosensory complex guides spindle orientation. *Nat. Commun.* 7:10899 doi: 10.1038/ncomms10899 (2016).

## Supplementary Material

Supplementary Information Supplementary Figures 1-8
