# Supplementary material for: A ligand-independent integrin β1 mechanosensory complex guides spindle orientation
Source: Nat Commun. 2016 Mar 8;7:10899. doi: 10.1038/ncomms10899 (PMC4786777; doi:10.1038/ncomms10899)
Supplement: Supplementary Information — Supplementary Figures 1-8 [file ncomms10899-s1.pdf]

Supplementary Information

*Supplementary Figures and Figure Legends*

## Supplementary Figure 1

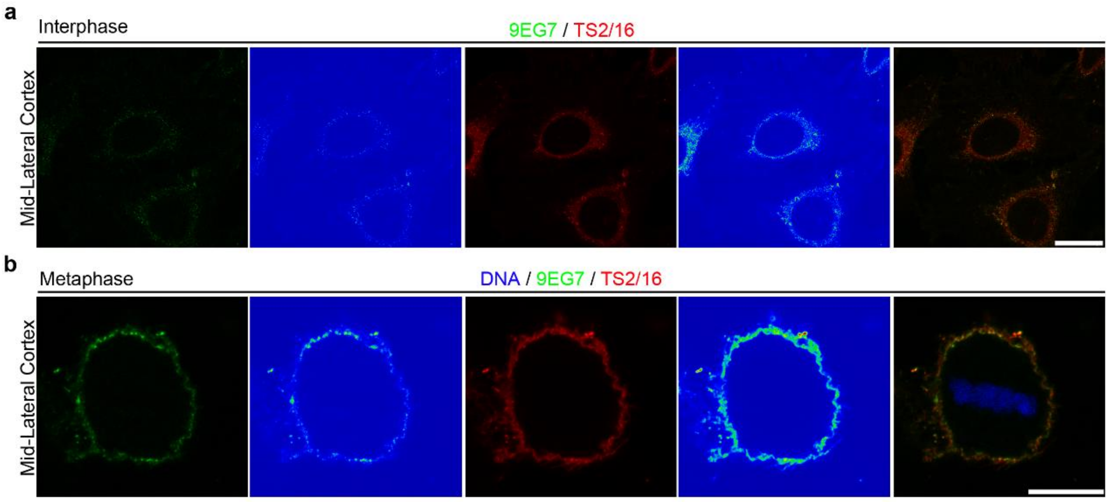


**Active integrin β1 is localized at the lateral cortex of mitotic cells.**

Optical sections, color intensity coded images and merged images at the plane of the mid-lateral cortex of interphase (**a**) and metaphase (**b**) cells stained for total integrin β1 (TS2/16 antibody), active integrin β1 (9EG7 antibody) and TO-PRO for DNA labeling. Scale bars: (**a**) 20 µm, (**b**) 10 µm.

**Supplementary Figure 2**

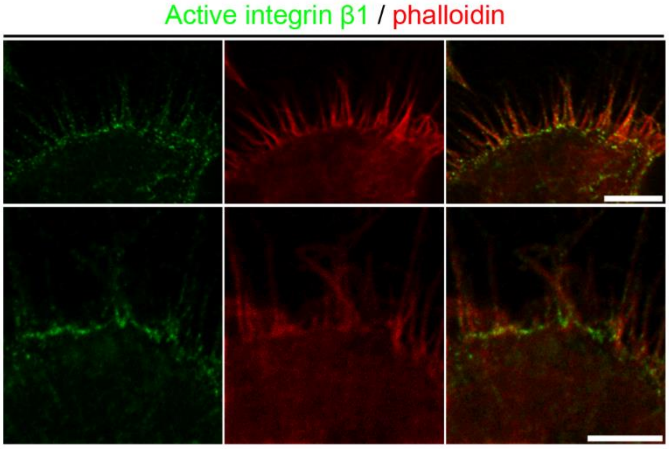


**Integrin β1 is activated at the regions where the RFs merge with the cell cortex.**

Thin optical sections and close-ups of metaphase HeLa cells stained for active integrin β1 (HUTS-21) and actin. Scale bars: 5 μm.

**Supplementary Figure 3**

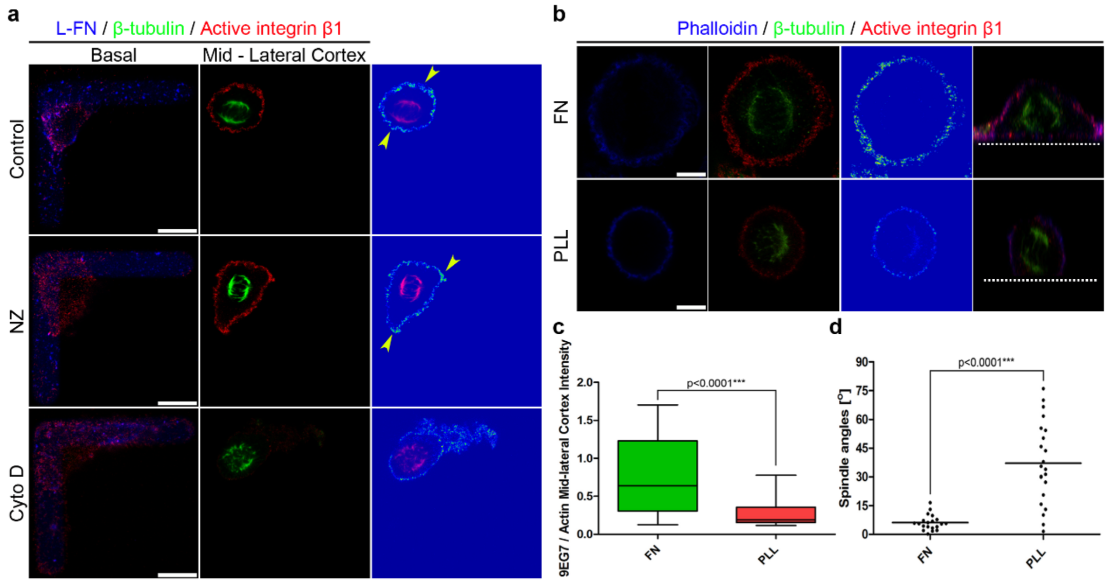


**Integrin β1 activation depends on the presence of RFs.**

(**a**) Optical sections at the cell-ECM interface and at the plane of the spindle of Control, NZ treated and Cyto D treated cells seeded on L-FN microprints. Yellow arrowheads indicate the cortical polarity of active integrin β1. (**b**) Optical sections, color intensity coded images and side views of cells seeded on FN or PLL, stained for β-tubulin, active β1 (9EG7) and actin. (**c**) Box-plot of the ratio of the intensity of mid-lateral cortical active integrin β1 to the intensity of cortical actin of metaphase cells seeded on FN and PLL. Mean ± SEM: FN 0.7494 ± 0.1131, n=20; PLL 0.2628 ± 0.03568, n=22; p<0.0001*** analyzed by t-test; n, number of metaphase cells, two independent experiments. (**d**) Scatter plot of the substrate to spindle angles of the cells analyzed in **c**. Mean ± SEM: FN 6.085 ± 0.8938°, n=20; PLL 37.07 ± 4.551°, n=22; p<0.0001*** analyzed by t-test; n, number of metaphase cells, two independent experiments. Scale bars: (**a**) 10 µm, (**b**) 5 µm.

## Supplementary Figure 4

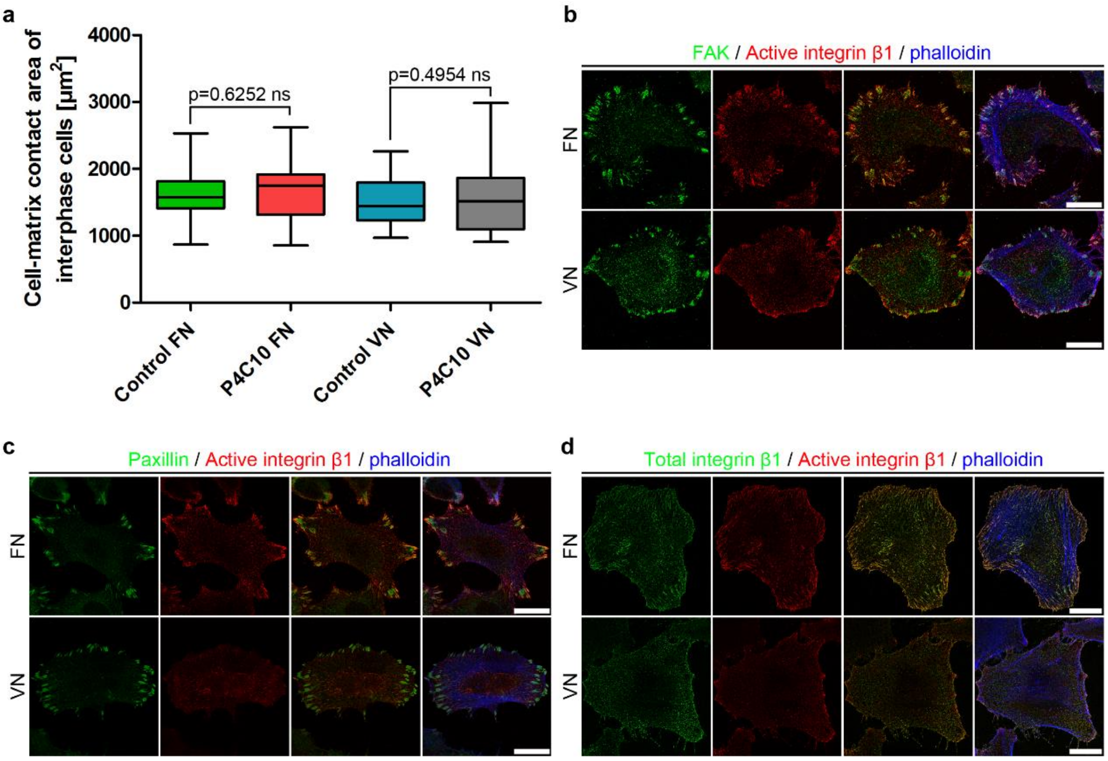


**Adhesion of HeLa cells on VN does not rely on integrin β1.**

(**a**) Box-plot of the cell-matrix contact area of interphase control cells or P4C10 treated cells seeded on FN or VN. Mean ± SEM: Control FN 1602 ± 85.95 µm$^2$, n=20; P4C10 FN 1667 ± 99.87 µm$^2$, n=20; Control VN 1499 ± 74.48 µm$^2$, n=20; P4C10 VN 1599 ± 124.9 µm$^2$, n=20; p values were calculated by t-test; n, number of metaphase cells, two independent experiments. (**b**, **c**, **d**) Optical sections of HeLa cells seeded on FN or VN and stained for active integrin β1 (9EG7), actin and the FA proteins FAK (**b**), Paxillin (**c**) or total integrin β1 (K20 antibody) (**d**). Scale bars: (**b-d**) 20 µm.

**Supplementary Figure 5**

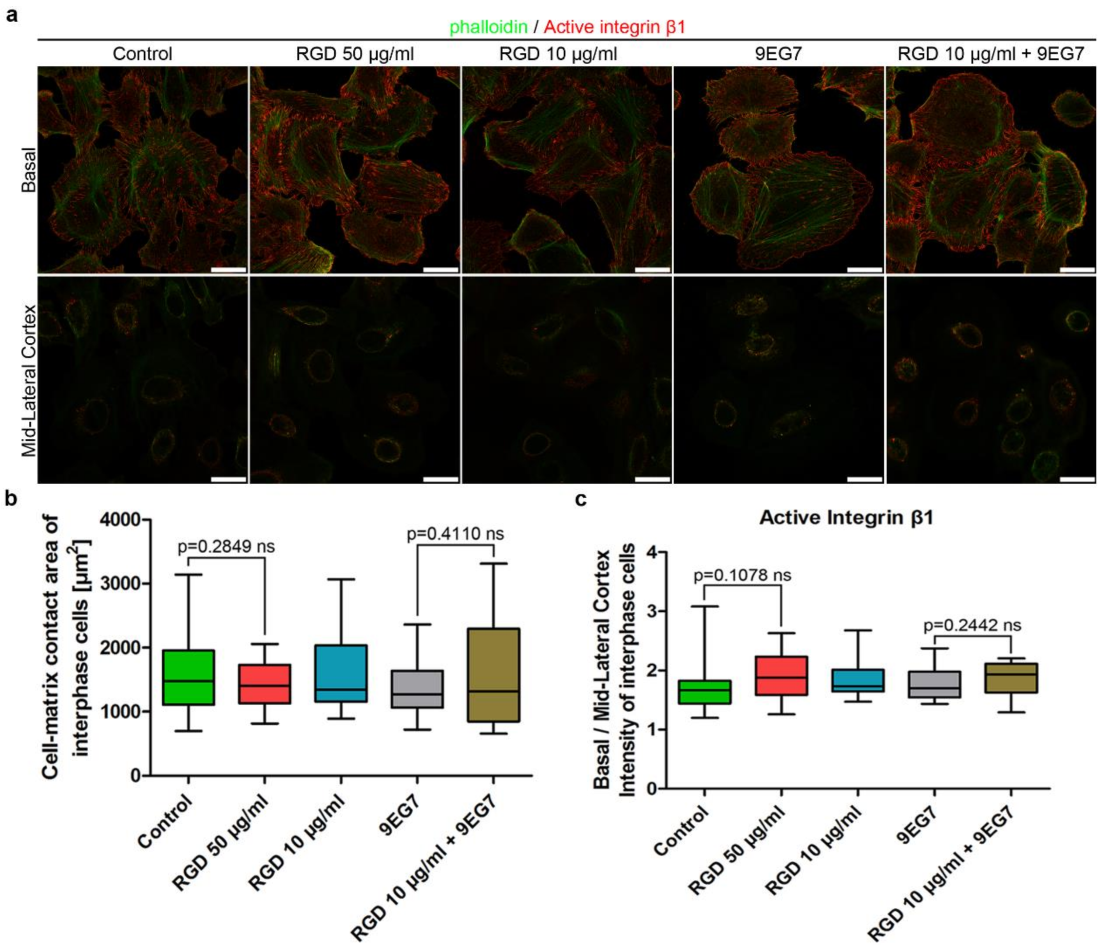


**RGD-mediated cortical integrin β1 overactivation does not affect cell-ECM adhesion or the distribution of active integrin β1 during interphase.**

(**a**) Optical sections at the cell-ECM interface and at the plane of the nucleus of interphase control cells, or cells treated as indicated and stained for actin and active integrin β1 (HUTS-21). (**b**) Box-plot of the cell-matrix contact area of interphase cells described in **a**. Mean ± SEM: Control 1591 ± 148.6 $\mu m^2$, n=20; RGD 50 µg/ml 1407 ± 80.36 $\mu m^2$, n=20; RGD 10 µg/ml 1553 ± 129.2 $\mu m^2$, n=20; 9EG7 1371 ± 95.35 $\mu m^2$, n=20; RGD 10 µg/ml + 9EG7 1539 ± 177.8 $\mu m^2$, n=20; p values were calculated by t-

test; n, number of interphase cells, two independent experiments. (**c**) Box-plot of the ratio of basal to mid-lateral cortex intensity of active integrin β1 of the cells described in **a**. Mean ± SEM: Control 1.713 ± 0.09153, n=20; RGD 50 µg/ml 1.918 ± 0.08440, n=20; RGD 10 µg/ml 1.850 ± 0.07315, n=20; 9EG7 1.768 ± 0.05489, n=20; RGD 10 µg/ml + 9EG7 1.868 ± 0.06438, n=20; p values were calculated by t-test; n, number of interphase cells, two independent experiments. Scale bars: (**a**) 20 µm.

**Supplementary Figure 6**

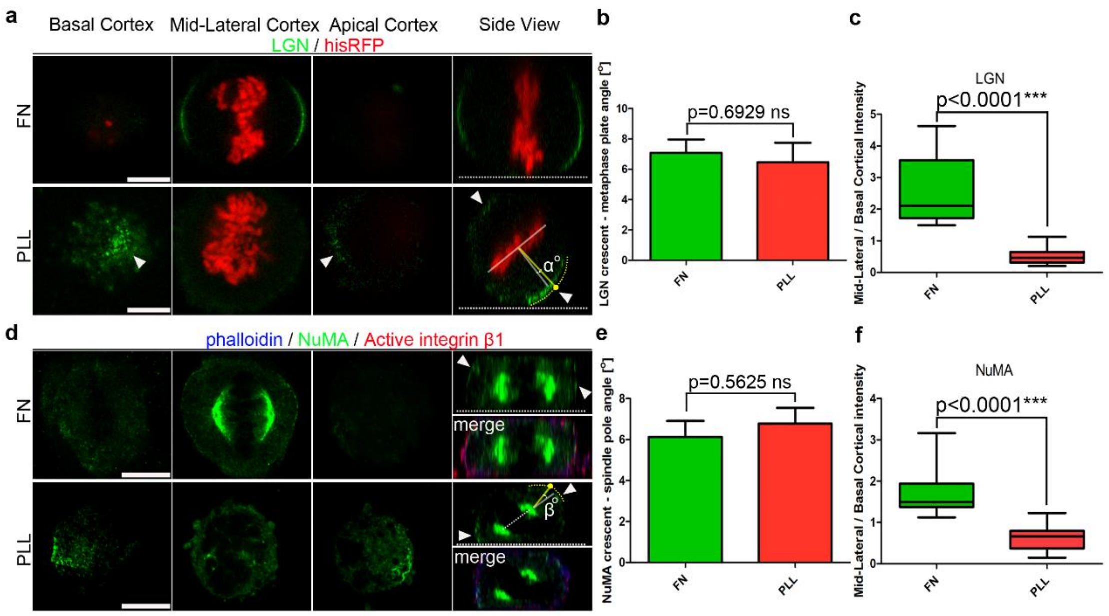


**LGN and NuMA cortical accumulation is not affected upon inhibition of integrin β1 signaling.**

(**a**) Z-stacks and side view of live metaphase HeLa cells expressing GFP-LGN and histone RFP seeded either on FN or PLL. White arrowheads indicate the cortical accumulation of LGN. (**b**) Plot for the cells described in **a** of the angle α formed between the line extending from the metaphase plate perpendicularly to the cell cortex and the line extending from the center of the LGN cortical crescent towards the center of the metaphase plate. Mean ± SEM: FN 7.083 ± 0.8801°, n=12; PLL 6.458 ± 1.290°, n=12; p values were calculated by t-test; n, number of metaphase cells, two independent experiments. (**c**) Plot of the ratio of mid-lateral cortical intensity to basal cortical intensity of LGN for the cells indicated in **a**. Mean ± SEM: FN 2.565 ± 0.2997, n=12; PLL 0.5078 ± 0.07286, n=12; p values were calculated by t-test; n, number of metaphase cells, two independent experiments. (**d**) Z-stacks, side view

and a merged side view of metaphase HeLa cells stained for NuMA, actin and active integrin β1 (9EG7) seeded either on FN or PLL. White arrowheads indicate the cortical accumulation of NuMA. (**e**) Plot for the cells indicated in **d** of the angle β formed between the line connecting the spindle poles and extending towards the proximal cortex and the line extending from the center of the NuMA cortical crescent towards the center of the proximal spindle pole. Mean ± SEM: FN 6.132 ± 0.7805$^{o}$, n=19; PLL 6.779 ± 0.7690$^{o}$, n=24; p values were calculated by t-test; n, number of metaphase cells, two independent experiments. (**f**) Plot of the ratio of mid-lateral cortical intensity to basal cortical intensity of NuMA for the cells indicated in **d**. Mean ± SEM: FN 1.711 ± 0.1166, n=19; PLL 0.6237 ± 0.05880, n=25; p values were calculated by t-test; n, number of metaphase cells, two independent experiments. Scale bars: (**a, d**) 10 µm.

**Supplementary Figure 7**

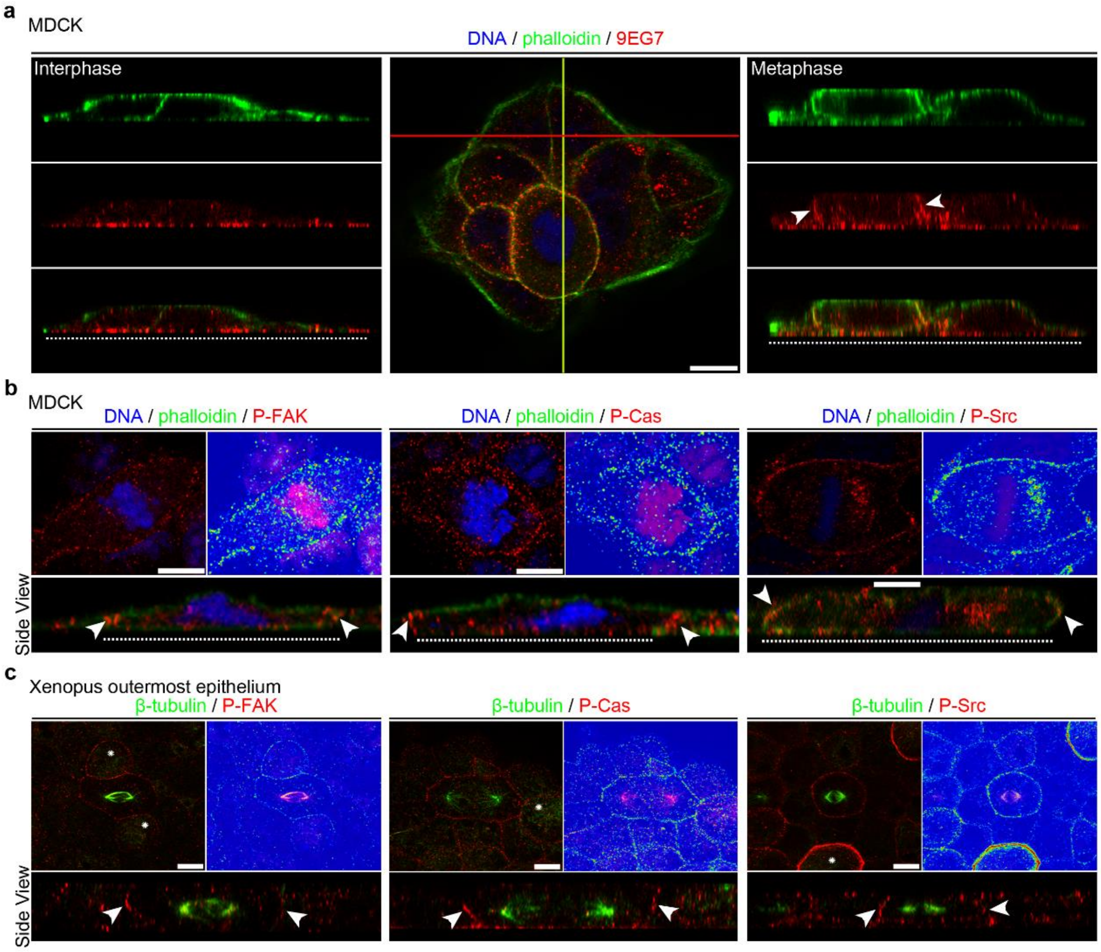


**Conservation of the formation of the CMC in several cell types.**

(**a**) Optical section and side views of interphase (section at the red line) and mitotic (section at the yellow line) cells in the same colony of MDCK cells. Cells were stained with the 9EG7 antibody, phalloidin and TO-PRO. Cell-ECM interface is represented by the white dashed line. Arrowheads show integrin β1 activation at the lateral cortex of the mitotic cell. (**b**) Optical sections at the spindle plane, color intensity coded images and side views of representative MDCK cells stained with phalloidin, TO-PRO and antibodies against phosphorylated FAK, Cas or Src. White arrowheads show

enrichment of the phosphorylated proteins at the lateral cortex of mitotic cells, white dashed line represents the ECM. (**c**) Optical sections at the plane of the spindle, color intensity coded images and side views of representative epithelial cells of the superficial cell layer of the Xenopus embryonic epidermis. Staining for β-tubulin and the phosphorylated forms of FAK, Cas and Src reveals elevated levels of the above proteins on the cortex of mitotic cells (white stars indicate the ciliated cells). The white arrowheads show enrichment of the phosphorylated proteins at the lateral cortex of mitotic cells. Scale bars: (**a-c**) 10 µm.

**Supplementary Figure 8**

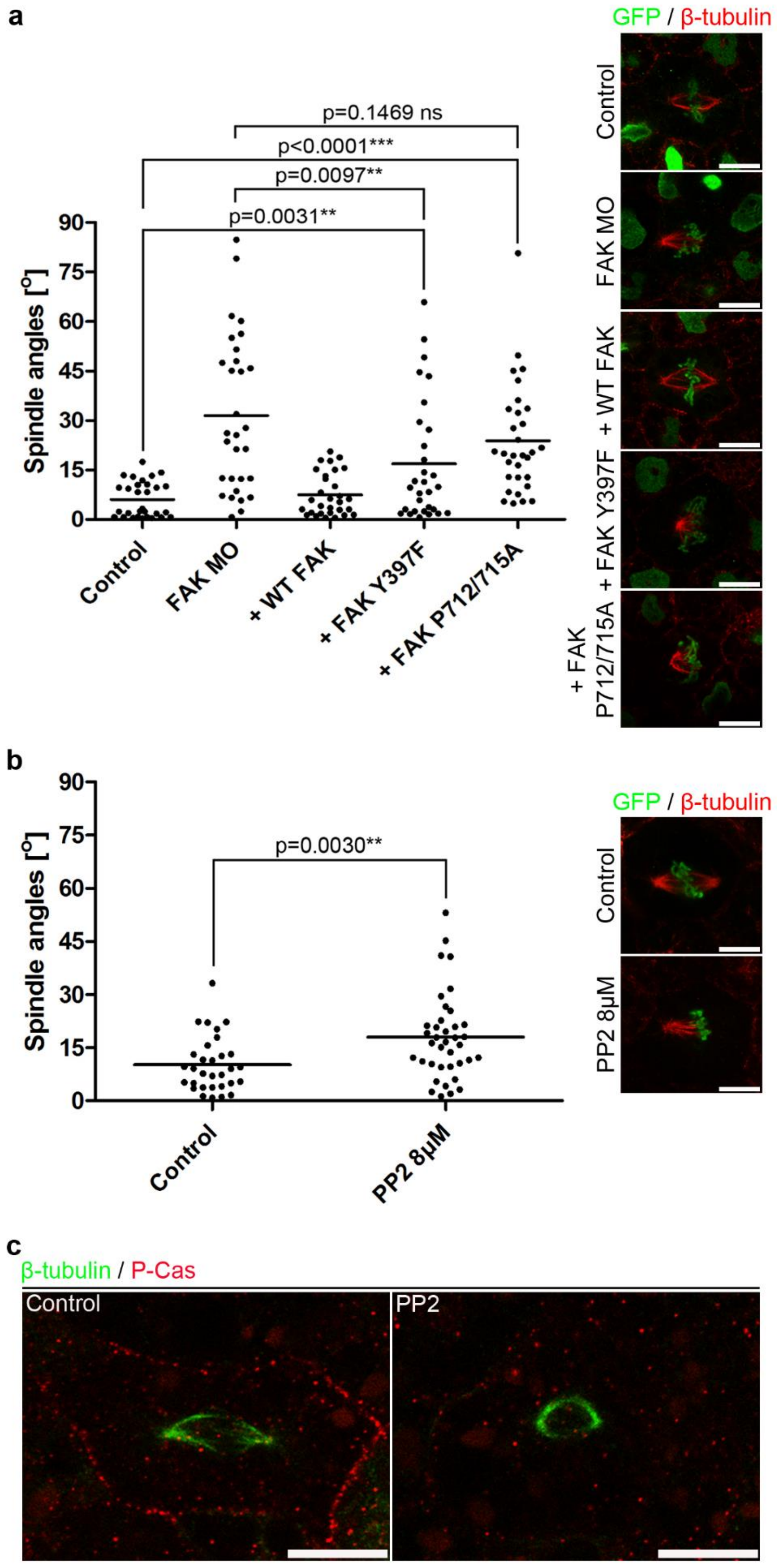

**Interactions between the members of the CMC are required to orient the mitotic spindle in the cells of Xenopus outermost epithelium.**

(**a**) Representative top views of mitotic control cells (injected with histone GFP) or cells injected with histone GFP, FAK MO and the indicated FAK constructs of the Xenopus outermost epithelium and scatter plot of the apical surface to spindle angles of these cells. Mean ± SEM: Control 6.033 ± 0.9885°, n=30; FAK MO 31.46 ± 4.301°, n=30; FAK MO + WT FAK 7.442 ± 1.176°, n=30; FAK MO + FAK Y397F 16.85 ± 3.358°, n=30; FAK MO + FAK P712/715A 23.81 ± 2.979°, n=31; p values were calculated by t-test; n, number of metaphase cells, two independent experiments. Cells were stained with β-tubulin and GFP antibodies. (**b**) Representative top views of mitotic histone GFP injected control or PP2 treated cells of the Xenopus outermost epithelium and a scatter plot of the apical surface to spindle angles of these cells. Mean ± SEM: Control 10.17 ± 1.389°, n=31; PP2 17.93 ± 1.972°, n=38; p=0.0030** analyzed by t-test; n, number of metaphase cells, two independent experiments. Cells were stained with β-tubulin and GFP antibodies. (**c**) Optical sections at the plane of the spindle of mitotic control or PP2 treated cells of the Xenopus outermost epithelium stained for β-tubulin and P-Cas antibodies. Scale bars: (**a-c**) 10 µm.
